# Bioorthogonal Click‐to‐Disassemble Phthalocyanine Nanoassemblies for Precise Chemo‐Photodynamic Therapy against Hypoxic Tumors

**DOI:** 10.1002/advs.78624

**Published:** 2026-11-09

**Authors:** Jiaxin Shen, Evelyn Y. Xue, Caixia Yang, Peter Chi Keung Cheung, Dennis K. P. Ng

**Affiliations:** ^1^ Department of Chemistry The Chinese University of Hong Kong Hong Kong China; ^2^ Department of Biomedical Engineering The Chinese University of Hong Kong Hong Kong China; ^3^ School of Life Sciences The Chinese University of Hong Kong Hong Kong China

**Keywords:** bioorthogonal chemistry, hypoxia, photodynamic therapy, phthalocyanines, self‐assembly

## Abstract

Photodynamic therapy (PDT) offers precise spatiotemporal control over cancer treatment but remains hampered by nonspecific photosensitizer activation, oxygen dependence, and some other limitations. Herein, we report a bioorthogonal “click‐to‐disassemble” strategy that enables tumor‐selective activation of self‐assembled phthalocyanine nanoassemblies for chemo‐photodynamic therapy against hypoxic tumors. An amphiphilic zinc(II) phthalocyanine bearing three triethylene glycol chains and a tetrazine‐functionalized substituent spontaneously self‐assembles into photodynamically silent nanoparticles through aggregation‐induced quenching while simultaneously encapsulating the hypoxia‐activated prodrug tirapazamine (TPZ). A bicyclo[6.1.0]non‐4‐yne (BCN) activator installed with an epidermal growth factor receptor (EGFR)‐targeting peptide through a cathepsin B‐cleavable linker selectively delivers the bioorthogonal trigger to EGFR‐overexpressing cancer cells. Intracellular cleavage of the linker releases a small hydrophilic BCN moiety, which reacts rapidly with the tetrazine unit through an inverse electron‐demand Diels–Alder reaction, converting the amphiphilic photosensitizer into a hydrophilic product that disrupts the nanoassemblies. This process simultaneously restores photodynamic activity and releases TPZ, which is activated by PDT‐enhanced tumor hypoxia to achieve cooperative chemotherapy. The bioorthogonally triggered activation minimizes off‐target phototoxicity while markedly enhancing tumor‐selective therapeutic efficacy in vitro and in vivo. This work establishes bioorthogonal disassembly as a general strategy for spatiotemporal activation of self‐assembled therapeutic nanomaterials and expands the toolbox of precision photomedicine.

## Graphical abstract

A bioorthogonal “click‐to‐disassemble” strategy enables precise activation of self‐assembled phthalocyanine nanoassemblies for chemo‐photodynamic therapy. EGFR‐targeted activator triggers IEDDA‐mediated disruption of the quenched assemblies, restoring fluorescence and ROS generation while releasing tirapazamine. PDT‐induced oxygen depletion further activates tirapazamine, converting tumor hypoxia into a therapeutic advantage and enhancing antitumor efficacy.

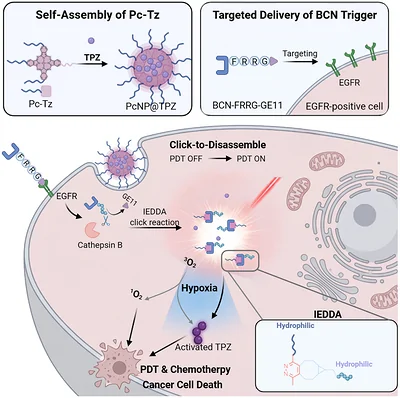

## 1 Introduction

Photodynamic therapy (PDT) has emerged as a promising treatment modality for localized malignancies that utilizes light‐activated photosensitizers to generate cytotoxic reactive oxygen species (ROS) in the presence of molecular oxygen [1, 2]. Since ROS are produced only within the irradiated region and possess short lifetimes and limited diffusion distances, PDT provides excellent spatiotemporal control over tissue damage while minimizing systemic toxicity. Nevertheless, its broader clinical application remains constrained by several interrelated limitations [3]. Many conventional photosensitizers lack intrinsic tumor selectivity and remain photodynamically active during circulation and tissue distribution, resulting in prolonged cutaneous photosensitivity and unintended phototoxicity. Moreover, the hydrophobic and highly conjugated structures of commonly used photosensitizers, including porphyrins and phthalocyanines, often lead to poor aqueous solubility, nonspecific aggregation, and suboptimal pharmacokinetic behavior. The therapeutic efficacy of PDT is further compromised by the hypoxic tumor microenvironment and rapid oxygen consumption during irradiation [4, 5]. Developing photosensitizing systems that can be delivered efficiently to tumors, remain inactive in normal tissues, and become selectively activated at the disease site therefore represents an important objective in precision photomedicine [6].

Self‐assembly of amphiphilic photosensitizers offers an attractive means of addressing several of these challenges [5, 7, 8]. By integrating hydrophobic chromophores with hydrophilic substituents, molecular photosensitizers can spontaneously organize into well‐defined nanoassemblies in aqueous media without requiring additional carrier materials. Such carrier‐free or carrier‐minimized systems can improve aqueous dispersibility and pharmacokinetic behavior, prevent premature leakage of the photosensitizing component, and provide hydrophobic domains for the co‐encapsulation of complementary therapeutic agents. Importantly, the close intermolecular packing and *π*–*π* interactions of the chromophores within these nanoassemblies commonly induce aggregation‐caused quenching, suppressing both fluorescence and ROS generation [9]. Although aggregation is generally detrimental to PDT, this otherwise unfavorable property can be exploited to construct activatable photosensitizers. Stimulus‐induced disassembly of these photodynamically silent nanoparticles subsequently separates the molecules, restores their excited‐state photochemistry, and activates ROS generation. The assembly state of the photosensitizer can thus serve as a molecular switch that directly controls its photodynamic function.

A variety of endogenous stimuli, including acidic pH, glutathione, enzymes, proteins, and nucleic acids, have been employed to trigger the disassembly of self‐assembled photosensitizer nanostructures [10]. However, the concentrations of these intrinsic stimuli often differ only modestly between tumors and normal tissues, resulting in limited specificity and undesirable background activation. Consequently, there remains a compelling need for activation mechanisms that operate independently of endogenous biochemical gradients while maintaining excellent selectivity within complex biological environments.

Bioorthogonal chemistry provides an attractive solution to this challenge. These abiotic reactions proceed rapidly and selectively under physiological conditions without perturbing endogenous biochemical processes, making them particularly suitable for precise molecular manipulation in living systems [11, 12]. Among the available bioorthogonal transformations, the inverse electron‐demand Diels–Alder (IEDDA) reaction between tetrazines and strained alkenes or alkynes exhibits exceptionally fast kinetics and excellent biocompatibility, and has become one of the most powerful ligation reactions for biomedical applications [13, 14]. Beyond biomolecular labeling and prodrug activation, bioorthogonal chemistry has recently emerged as an effective strategy for regulating supramolecular nanostructures through stimulus‐triggered disassembly. For example, Porte et al. reported a self‐assembled micellar nanosystem composed of an iminosydnone‐functionalized amphiphile, in which a tumor‐generated cyclooctyne triggered a bioorthogonal “click‐to‐release” reaction that selectively disassembled the nanostructures and released the encapsulated therapeutic cargo [15]. Likewise, Song et al. demonstrated that intracellular IEDDA reactions between tetrazine‐containing supramolecular assemblies and *trans*‐cyclooctene (TCO) derivatives disrupted intermolecular *π*–*π* interactions, leading to controlled dissociation of the assemblies inside living cells [16]. These elegant studies clearly demonstrate the potential of bioorthogonal chemistry to manipulate supramolecular organization with high spatial precision. Nevertheless, to the best of our knowledge, this concept has not yet been extended to the activation of self‐assembled photosensitizers for PDT. We envisioned that a bioorthogonal ligation capable of altering the molecular amphiphilicity of an assembled photosensitizer would trigger nanoparticle disassembly, restore its photodynamic activity, and simultaneously release therapeutic payloads in a single molecular event, thereby providing precise spatiotemporal control over chemo‐photodynamic therapy.

Herein, we present a bioorthogonal “click‐to‐disassemble” platform for targeted elimination of hypoxic tumors through cooperative chemo‐photodynamic therapy. As illustrated in Figure 1, the system is based on an amphiphilic zinc(II) phthalocyanine (ZnPc) bearing three hydrophilic triethylene glycol chains and a tetrazine‐functionalized hydrophobic substituent, which spontaneously self‐assembles into photodynamically inactive nanoparticles in aqueous media. The hydrophobic interior of the nanoparticles also accommodates the hypoxia‐activated prodrug tirapazamine (TPZ) [17]. To enable tumor‐selective bioorthogonal activation, a complementary bicyclo[6.1.0]non‐4‐yne (BCN) trigger was conjugated to the epidermal growth factor receptor (EGFR)‐targeting peptide GE11 with the sequence YHWYGYTPQNVI [18] through a cathepsin B‐cleavable FRRG linker [19]. Following receptor‐mediated uptake by EGFR‐overexpressing cancer cells, intracellular cleavage of the FRRG linker by lysosomal cathepsin B, which is also overexpressed in cancer cells, removes the bulky targeting peptide and exposes a less sterically hindered BCN‐bearing fragment. This fragment can access the hydrophobic region of the nanoassemblies and undergo rapid IEDDA ligation with the tetrazine‐functionalized phthalocyanine. The resulting increase in molecular hydrophilicity disrupts the amphiphilic balance of the photosensitizer, leading to disassembly of nanoparticles, restoration of photodynamic activity, and concomitant release of TPZ. Because TPZ is activated under hypoxic conditions generated during PDT [20], this sequential cascade couples bioorthogonal chemistry, activatable PDT, and hypoxia‐responsive chemotherapy within a single molecular platform. More broadly, this work establishes bioorthogonal modulation of molecular amphiphilicity as a general design principle for spatiotemporally controlled activation of self‐assembled therapeutic nanomaterials.

**FIGURE 1:**
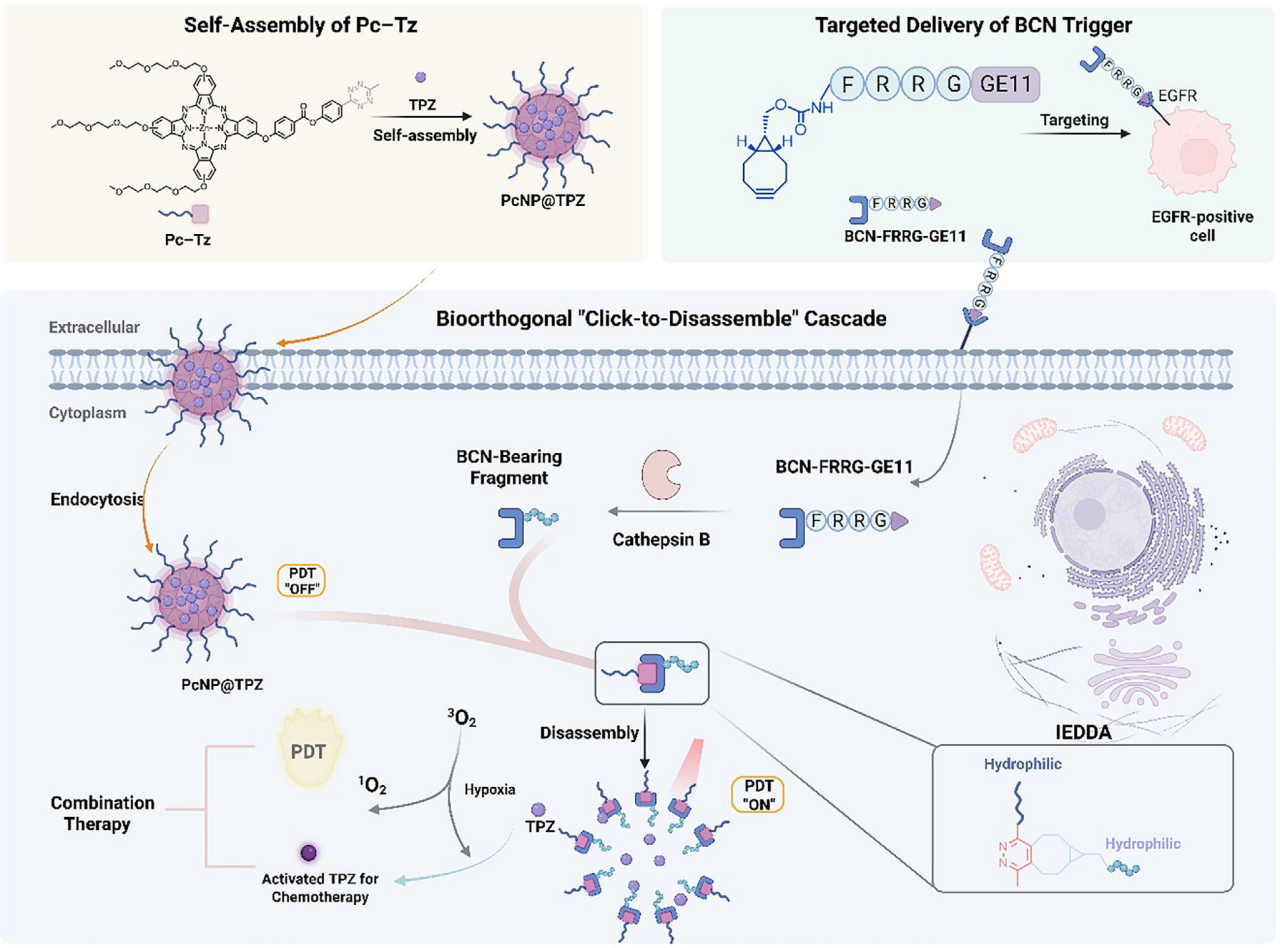
Schematic illustration of the fabrication of TPZ‐encapsulated phthalocyanine nanoassemblies, EGFR‐mediated uptake of BCN trigger, and the bioorthogonal “click‐to‐disassemble” mechanism for targeted chemo‐photodynamic therapy.

## 2 Results and Discussion

### 2.1 Chemical Synthesis, Nanoparticle Formation, and Characterization

For the self‐assembled photosensitizing system, ZnPc was selected as the building block due to its intense absorption in the far‐red visible region (λ_abs_ ≈ 680 nm, log ε > 5), efficient generation of ROS, high photostability, pronounced aggregation‐induced self‐quenching, and amenability to structural modification [21]. An amphiphilic scaffold (**Pc–Tz**) was designed, and its synthesis is outlined in Scheme S1. Treatment of triethylene glycol‐substituted phthalonitrile **1** with methyl benzoate‐modified phthalonitrile **2** in the presence of Zn(OAc)_2_ and 1,8‐diazabicyclo[5.4.0]undec‐7‐ene (DBU) in 1‐pentanol led to mixed cyclization of these precursors, and ZnPc **3** was isolated in 14%. As reported earlier [22], transesterification occurred with the solvent under this basic condition, converting the methyl into pentyl ester. Upon alkaline hydrolysis, the carboxy intermediate formed was coupled with tetrazine‐substituted phenol **4** to give the amphiphilic target **Pc–Tz**.

Owing to the combination of three hydrophilic triethylene glycol chains and a hydrophobic tetrazine‐substituted phenyl benzoate moiety, **Pc–Tz** spontaneously self‐assembled in deionized water containing 2% (v/v) dimethyl sulfoxide (DMSO) to form phthalocyanine nanoparticles, denoted as **PcNP**. The hydrodynamic diameter of the nanoparticles was determined to be approximately 126 nm by dynamic light scattering (DLS) (Figure 2a). The size was essentially unchanged in the dark at ambient temperature over a period of 6 days, reflecting the high stability of the nanoparticles (Figure S1a). Transmission electron microscopy (TEM) revealed that they are nearly spherical in shape with a size of ca. 110 nm (Figure S1b).

**FIGURE 2:**
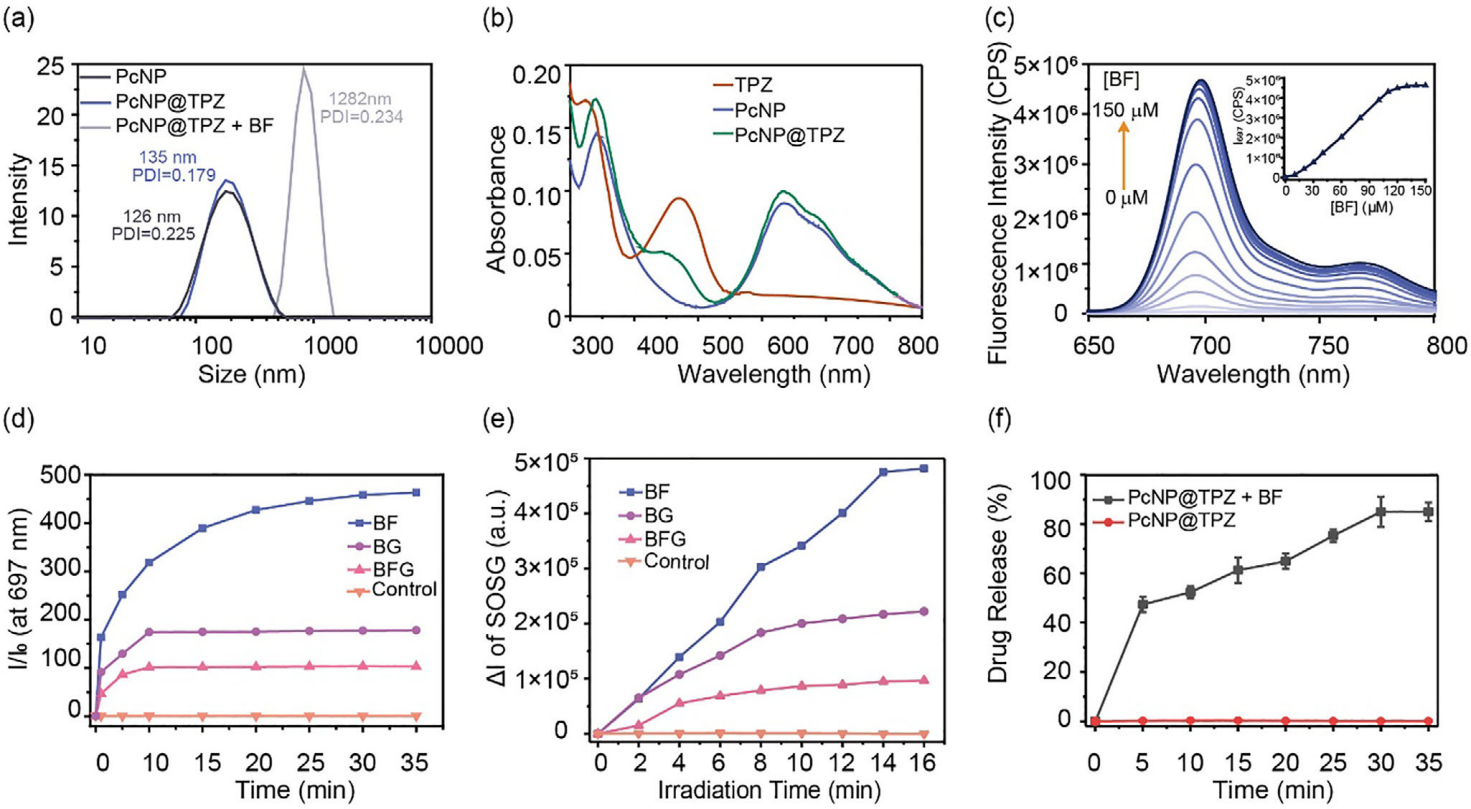
(a) Hydrodynamic diameter distributions of **PcNP** and **PcNP@TPZ** [with or without the treatment with **BF** (120 µm) for 35 min] in deionized water. The concentration of **Pc–Tz** was fixed at 5 µm. (b) Electronic absorption spectra of **PcNP** and **PcNP@TPZ** (at 1 µm of **Pc–Tz**) in deionized water and free TPZ in DMSO (15 µm). (c) Fluorescence spectra of **PcNP** (at 5 µM of **Pc–Tz**) after the treatment with different concentrations of **BF** (0–150 µm) in deionized water for 60 min. The inset shows the change in fluorescence intensity at 697 nm with the concentration of **BF**. (d) Change in fluorescence intensity at 697 nm of **PcNP** (at 5 µm of **Pc–Tz**) in deionized water in the absence and presence of **BF**, **BG**, or **BFG** (all at 120 µm) over a period of 35 min. (e) Change in fluorescence intensity of SOSG (10 µm) in deionized water containing **PcNP** (at 5 µm of **Pc–Tz**) with or without the treatment with **BF**, **BG**, or **BFG** (all at 120 µm) for 35 min. The mixtures were irradiated (λ > 610 nm) over a period of 16 min. (f) TPZ release profiles of **PcNP@TPZ** (at 5 µm of **Pc–Tz**) in the absence and presence of **BF** (120 µm) in deionized water.

In the presence of TPZ, the prodrug was encapsulated during the self‐assembly of **Pc–Tz**, giving **PcNP@TPZ**. After dialysis in deionized water for 12 h to remove the free TPZ and residual DMSO, the nanoparticles were characterized by DLS. As expected, the hydrodynamic diameter (ca. 135 nm) was slightly larger than that of **PcNP** (Figure 2a). Figure 2b shows the electronic absorption spectrum of **PcNP@TPZ** and those of free TPZ and **PcNP** for comparison. In addition to the characteristic ZnPc absorption, **PcNP@TPZ** displayed the diagnostic TPZ absorption band at 462 nm, confirming successful incorporation of the prodrug into the nanoassemblies. By using UV–vis spectroscopy, it was found that the molar ratio of **Pc–Tz** to TPZ was approximately 1:7 in **PcNP@TPZ**, and the encapsulation efficiency was determined to be 27%.

The amphiphilic balance of **Pc–Tz** is essential for maintaining the integrity of these nanoassemblies. We therefore reasoned that covalent modification of the tetrazine‐bearing hydrophobic segment through bioorthogonal ligation with a hydrophilic BCN derivative would perturb this balance, alter intermolecular packing, and promote disassembly or structural reorganization of the nanoparticles. Therefore, a series of BCN derivatives were designed and prepared. To enable tumor‐selective delivery of the bioorthogonal trigger, a BCN moiety was conjugated to the EGFR‐targeting peptide GE11 through the cathepsin B‐responsive tetrapeptide FRRG. The latter was introduced to promote the “click‐to‐release” process by reducing the bulkiness of the BCN moiety through selective cleavage of this sequence to detach the GE11 targeting group after internalization. For comparison, a BCN moiety was also conjugated to FRRG or directly to GE11. These three triggers BCN–FRRG, BCN–GE11, and BCN–FRRG–GE11, hereafter abbreviated as **BF**, **BG**, and **BFG**, respectively, were prepared as outlined in Scheme S2. Briefly, the FRRG, GE11, and FRRG‐GE11 peptide resins were prepared according to the standard fluorenylmethoxycarbonyl (Fmoc)‐based solid‐phase peptide synthesis protocol using rink amide resin as the solid support and the commercially available *N*‐α‐Fmoc‐protected amino acids as starting materials. After cleavage from the resin with a mixture of trifluoroacetic acid (TFA), triisopropylsilane (TIPS), and water (95:2.5:2.5, v/v/v), the *N*‐terminus of each peptide was coupled with 4‐nitrophenyl carbonate‐activated BCN **5** in the presence of *N,N*‐diisopropylethylamine (DIPEA) to give the corresponding conjugates. All peptides were purified by high‐performance liquid chromatography (HPLC) and characterized by matrix‐assisted laser desorption/ionization time‐of‐flight (MALDI–TOF) mass spectrometry (Figures S2–S4), while the purity and identity of **BF**, **BG**, and **BFG** were confirmed by liquid chromatography – electrospray ionization mass spectrometry (LC–ESI‐MS) (Figures S5–S7).

### 2.2 Bioorthogonally Triggered Structural Disruption, Photodynamic Activation, and Drug Release

Having prepared the tetrazine‐bearing phthalocyanine nanoassemblies and the complementary BCN derivatives, we first investigated whether the IEDDA reaction could disrupt the nanoassemblies and restore the fluorescence and photosensitizing properties of **Pc–Tz** in aqueous solution. DLS was first used to monitor the change in hydrodynamic diameter of **PcNP@TPZ** upon addition of **BF** in water (Figure 2a). The value was largely increased from ca. 135 nm to over 1200 nm, indicating extensive disruption and reorganization of the original nanostructure following the tetrazine–BCN reaction, with the resulting species likely undergoing secondary association in water. The recovery of **Pc–Tz** fluorescence upon addition of the BCN derivatives to **PcNP** was then studied. As shown in Figure 2c, upon treatment for 60 min, the fluorescence intensity increased with the concentration of **BF** up to approximately 120 µm, suggesting that maximal activation was achieved under these conditions. The kinetics of fluorescence restoration were then examined in the absence or presence of **BF**, **BG**, or **BFG**, each at 120 µm. In the absence of a BCN derivative, the fluorescence of **PcNP** remained negligible throughout the 35‐min observation period, confirming that the nanoparticles were stable and persistently quenched in water (Figure S8a). In contrast, addition of **BF** induced a rapid increase in emission at 697 nm (Figure S8b). The fluorescence intensity increased by approximately 160‐fold within the first min and by approximately 460‐fold after 35 min, demonstrating rapid and efficient dequenching following the IEDDA reaction. **BG** and **BFG** also restored the fluorescence of **PcNP** (Figure S8c,d), but considerably more slowly. The difference as depicted in Figure 2d is plausibly attributable to the size of the peptide components attached to BCN. Compared with FRRG, GE11 and FRRG–GE11 impose greater steric and diffusional barriers to BCN access to the tetrazine groups within the nanoassemblies. These results support the proposed rationale that removal of the bulky GE11‐containing segment from **BFG** should facilitate bioorthogonal activation in the intracellular environment.

To obtain direct molecular‐level evidence for the proposed bioorthogonal transformation of the ZnPc building block, **PcNP** in deionized water (5 µm) was treated with **BF** (120 µm) at room temperature for 35 min, and the resulting mixture was analyzed by MALDI–TOF mass spectrometry. An intense signal at *m*/*z* 2050.814 was detected (Figure S9), which is in excellent agreement with the value calculated for the molecular ion of the ligation adduct of **Pc–Tz** and **BF** (*m*/*z* 2050.802). It unambiguously confirms that the tetrazine unit of **Pc–Tz** undergoes IEDDA ligation with the BCN moiety of **BF** to covalently install a hydrophilic peptide fragment onto the photosensitizer, triggering the disassembly of the nanoparticles.

We subsequently evaluated whether fluorescence recovery was accompanied by restoration of the photosensitizing activity of **Pc–Tz**. Singlet oxygen generation was examined in water using Singlet Oxygen Sensor Green (SOSG) as the fluorescent probe [23]. Without pre‐treatment with a BCN activator, the change in SOSG fluorescence was negligible (Figure S10a), confirming that singlet oxygen generation by the assembled **PcNP** was strongly suppressed. After the treatment with **BF** for 35 min, light irradiation of **PcNP** (λ > 610 nm) produced a pronounced time‐dependent increase in SOSG fluorescence at 530 nm, indicating efficient restoration of singlet oxygen generation (Figure S10b). **BG** and **BFG** also enhanced SOSG fluorescence, but both the rate and extent of activation were reduced compared with those observed for **BF** (Figure S10c,d), consistent with the corresponding fluorescence‐dequenching kinetics. A quantitative comparison of the different conditions is presented in Figure 2e. Collectively, these results demonstrate that the tetrazine–BCN ligation not only alters the assembly state of the nanoparticles but also restores the excited‐state processes required for photodynamic action.

The bioorthogonally triggered release of TPZ from **PcNP@TPZ** was then assessed by dialysis after treatment with **BF**. When **PcNP@TPZ** (at 5 µm of **Pc–Tz**) was incubated with **BF** (120 µm), the cumulative release of TPZ increased progressively with time, reaching approximately 80% within 30 min (Figure 2f). In contrast, negligible release was observed in the absence of **BF**. These findings indicate that bioorthogonal reaction‐induced disruption of the phthalocyanine nanoassemblies simultaneously restores the photodynamic activity of **Pc–Tz** and liberates the encapsulated hypoxia‐activated prodrug. Thus, the IEDDA reaction functions as a common molecular trigger that coordinates photosensitizer activation and drug release.

### 2.3 Bioorthogonal Activation of PcNP in Cancer Cells

Having established that the tetrazine–BCN ligation efficiently disrupted the phthalocyanine nanoassemblies and restored the photophysical properties of **Pc–Tz** in solution, we further investigated whether this bioorthogonal activation strategy could operate in living cells. A549 human lung adenocarcinoma cells, which overexpress both EGFR and cathepsin B [24], were selected as the primary model. Cells were first pre‐incubated with various concentrations of the bioorthogonal activator **BFG** (0–120 µm) for 4 h to allow receptor‐mediated internalization and intracellular enzymatic cleavage, followed by incubation with **PcNP** (at 5 µm of **Pc–Tz**) for 1 h. Confocal fluorescence microscopy revealed negligible intracellular fluorescence in the absence of **BFG**, indicating that the self‐assembled **PcNP** remained largely quenched after cellular uptake (Figure 3a). In contrast, pretreatment with **BFG** produced a pronounced and concentration‐dependent increase in **Pc–Tz** fluorescence, demonstrating efficient intracellular activation of the photosensitizer. Quantitative flow cytometric analysis confirmed this trend, showing a progressive increase in fluorescence intensity with increasing **BFG** concentration (Figure 3b). These observations indicate that the bioorthogonal reaction can effectively restore the emissive properties of the self‐quenched nanoassemblies within living cells.

**FIGURE 3:**
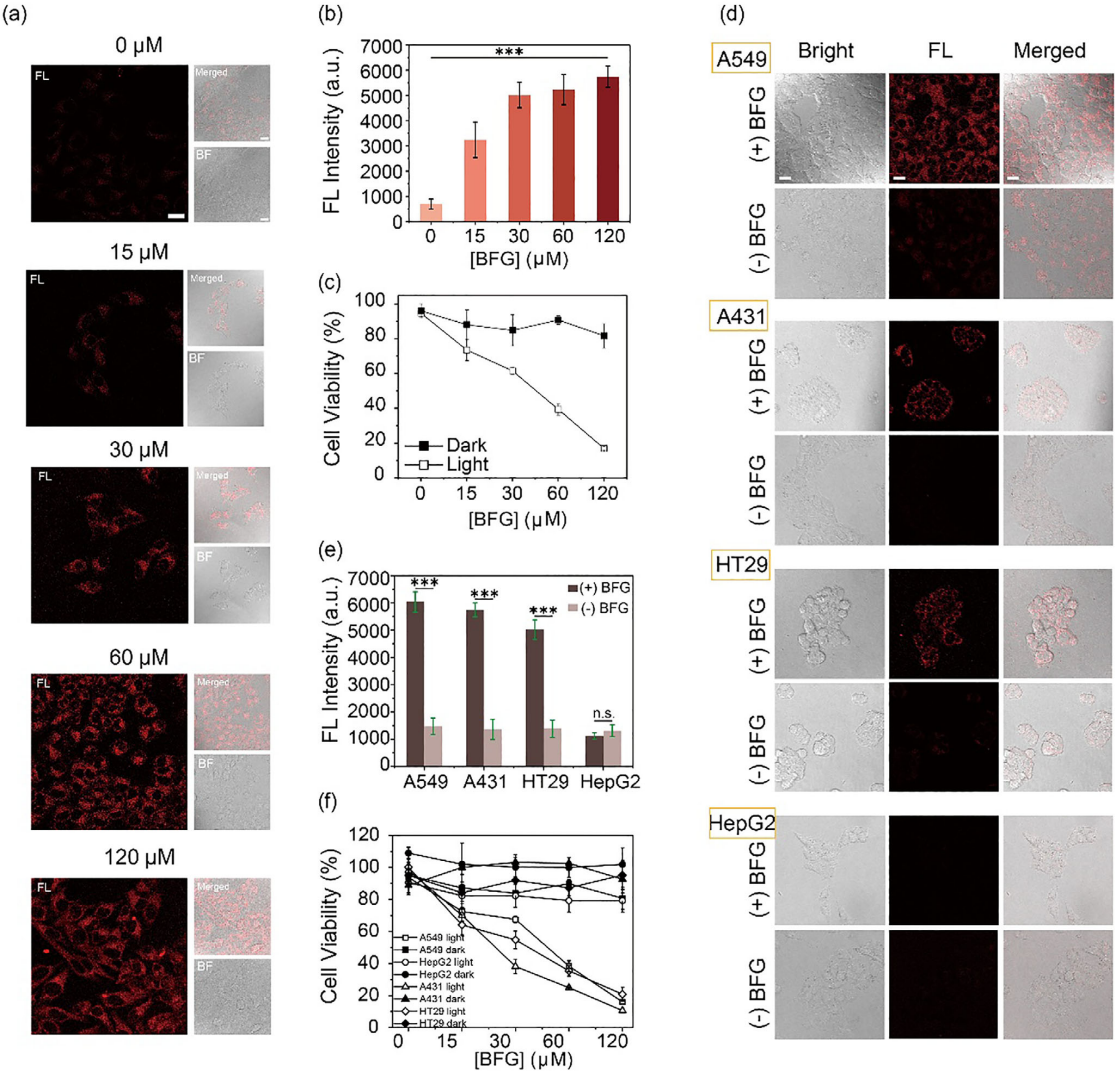
(a) Confocal microscopic images of A549 cells after incubation with **PcNP** (at 5 µm of **Pc–Tz**) for 1 h with or without pre‐incubation with different concentrations of **BFG** (0–120 µm) for 4 h. Scale bar: 20 µm. (b) Intracellular fluorescence intensities under the above conditions determined by flow cytometry. Data are reported as mean ± standard deviation (SD) of three independent experiments. (c) Cytotoxicity of **PcNP** (at 5 µm of **Pc–Tz**) triggered by different concentrations of **BFG**, both in the dark and upon light irradiation (λ > 610 nm, 23 mW cm^−2^) for 10 min. Data are reported as mean ± standard error of the mean (SEM) of three independent experiments, each performed in quadruplicate. (d) Confocal microscopic images of A549, A431, HT29, and HepG2 cells after incubation with **PcNP** (at 5 µm of **Pc–Tz**) for 1 h with or without pre‐incubation with **BFG** (120 µm) for 4 h. Scale bar: 20 µm. (e) Intracellular fluorescence intensities under the above conditions determined by flow cytometry. Data are reported as mean ± SD of three independent experiments. (f) Dark and photo‐cytotoxicity of **PcNP** triggered by **BFG** under the above conditions. Data are reported as mean ± SEM of three independent experiments, each performed in quadruplicate. Statistical significance was determined using a two‐tailed Student's *t*‐test (***p < 0.001).

To demonstrate the role of cathepsin B in the activation process, we performed a cathepsin B inhibition experiment using the cell‐permeable, selective inhibitor CA‐074Me in A549 cells. Cells were pretreated with CA‐074Me (25 µm, 2 h), followed by sequential incubation with **BFG** (120 µm, 4 h) and **PcNP** (5 µm, 1 h) as mentioned above. As expected, the intracellular fluorescence was largely reduced compared with the non‐CA‐074Me‐treated group, and the intensity was comparable with that of the baseline control group, i.e., cells incubated with **PcNP** alone (Figure S11). The marked attenuation of fluorescence recovery in the CA‐074Me‐treated group confirms that intracellular activation of the system is largely dependent on cathepsin B activity, consistent with our proposed “click‐to‐disassemble” mechanism in which enzymatic cleavage of the FRRG sequence removes the bulky GE11 segment and thereby facilitates the subsequent bioorthogonal activation.

The cytotoxicity of **PcNP** was further examined upon the treatment with **BFG** by MTT assay. As shown in Figure 3c, cell viability remained essentially unchanged in the absence of light irradiation, indicating that both **PcNP** and **BFG** exhibited negligible dark cytotoxicity over the investigated concentration range (5 µm of **Pc–Tz** and up to 120 µm **BFG**), confirming their excellent biocompatibility prior to photoactivation. Following irradiation (λ > 610 nm), however, cell viability decreased progressively with increasing **BFG** concentration. These results further confirm that the nanoassemblies are disassembled upon interactions with the trigger, restoring the photocytotoxicity of **Pc–Tz**.

To investigate the cell selectivity of this activation process, another two EGFR‐positive cell lines, namely A431 human epidermoid carcinoma cells and HT29 human colorectal adenocarcinoma cells [25], as well as an EGFR‐negative cell line, namely HepG2 human hepatocellular carcinoma cells [24], were also used for the study. Robust fluorescence activation was observed exclusively in the EGFR‐positive cells following **BFG** pretreatment, whereas the fluorescence signal was hard to detect in HepG2 cells (Figure 3d,e). Without pretreatment with **BFG**, all the cell lines exhibited negligible fluorescence. Consistent with the imaging results, pronounced photocytotoxicity was induced only in the EGFR‐positive cell lines after pretreatment with **BFG** followed by light irradiation, while HepG2 cells remained largely unaffected (Figure 3f). These findings demonstrate that bioorthogonal activation requires efficient cellular delivery of the BCN trigger through GE11‐mediated EGFR recognition, thereby conferring excellent cellular selectivity.

For comparison, **BG**, which lacks the cathepsin B‐responsive linker FRRG, was also used as an activator under the same conditions. Although both the intracellular fluorescence intensity (Figure S12a,b) and photocytotoxicity (Figure S12c–f) increased to some extent with the concentration of **BG**, the activation efficiency was significantly lower than that of **BFG**. The reduced activation efficiency is consistent with the bulky GE11 peptide limiting access of the BCN group to the tetrazine moieties embedded within the hydrophobic nanoassemblies. In contrast, intracellular cleavage of the FRRG linker in **BFG** is expected to remove the sterically demanding GE11‐containing segment, generating a significantly smaller BCN‐bearing species that can more readily penetrate the nanoassemblies and undergo the IEDDA reaction, promoting the disassembly and activation process.

Collectively, these studies establish a sequential activation cascade for the present system. EGFR‐mediated endocytosis first enriches the BCN precursor in target cells; intracellular cathepsin B‐mediated hydrolysis enhances the accessibility of the BCN trigger; and the subsequent tetrazine–BCN ligation disrupts the amphiphilic balance of **Pc–Tz**, triggering the disassembly of nanoassemblies and restoring both fluorescence and photodynamic activity. This multistage activation mechanism provides substantially higher cellular specificity than conventional stimulus‐responsive photosensitizers that rely solely on endogenous biochemical markers.

### 2.4 Bioorthogonal Activation of ROS Generation and Hypoxia‐Responsive Chemotherapy

A major limitation of conventional PDT is its dependence on molecular oxygen. During PDT, oxygen is continuously consumed to generate ROS, progressively aggravating tumor hypoxia and consequently limiting sustained ROS production. We reasoned that this intrinsic drawback could instead be exploited therapeutically by coupling PDT with the hypoxia‐activated prodrug TPZ, which induces free radical‐mediated oxidative DNA damage under hypoxia and thus can selectively kill hypoxic tumor cells [17]. Following bioorthogonal activation and disassembly of the nanoassemblies, the photosensitizer **Pc–Tz** would restore ROS generation upon irradiation while simultaneously consuming intracellular oxygen. The resulting hypoxic microenvironment was expected to activate the co‐released TPZ, thereby establishing a cascade in which PDT and hypoxia‐responsive chemotherapy mutually reinforce one another. To validate this concept, we investigated intracellular ROS production, hypoxia generation, and the combined cytotoxicity of **PcNP@TPZ** after bioorthogonal activation.

The restoration of the photodynamic activity was verified by monitoring intracellular ROS generation using 2’,7’‐dichlorodihydrofluorescein diacetate (H_2_DCFDA), which is hydrolyzed upon internalization and converts into highly fluorescent dichlorofluorescein (DCF) by intracellular ROS [26]. As expected, negligible DCF fluorescence was observed in the absence of **BFG** and/or light irradiation (Figure 4a, upper row), indicating that the intact **PcNP@TPZ** remained photodynamically inactive and the requirement of light for activation. In contrast, cells pretreated with **BFG** exhibited intense green fluorescence after irradiation, corresponding to an approximately eight‐fold increase in ROS production (Figure 4b).

**FIGURE 4:**
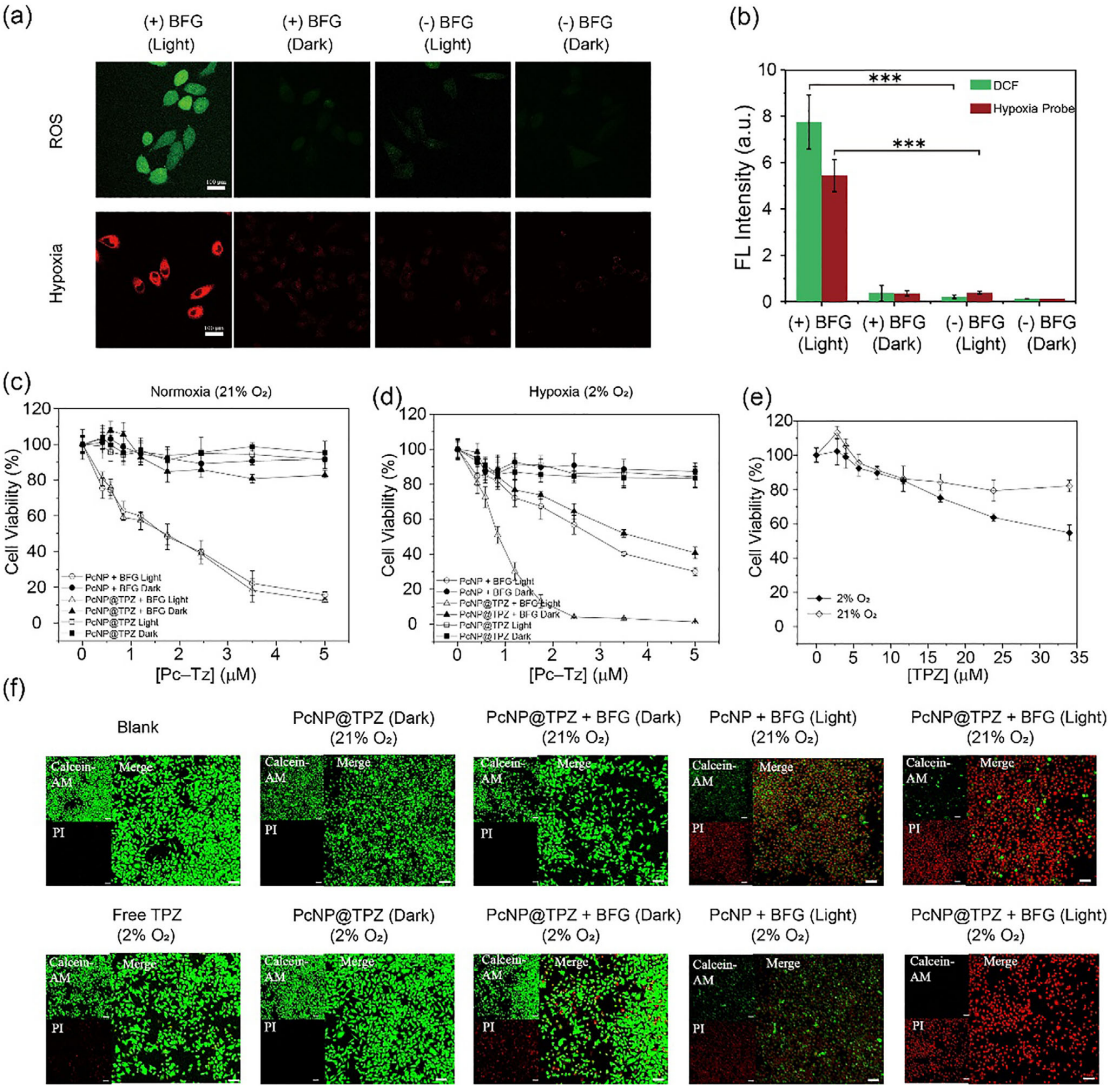
(a) Intracellular ROS (top) and hypoxia (bottom) levels in A549 cells after various treatments. Cells were incubated with **PcNP@TPZ** (at 5 µm of **Pc–Tz**) for 1 h with or without pretreatment with **BFG** (60 µm) for 4 h. For ROS detection, the cells were incubated with H_2_DCFDA (10 µm) for 30 min with or without further light irradiation (λ > 610 nm, 23 mW cm*^−^* ^2^) for 3 min. For hypoxia detection, the cells were incubated with Image‐iT Red Hypoxia Probe (15 µm) for 5 min after the above dark or light treatment. Scale bar: 100 µm. (b) Quantification of the fluorescence intensities of DCF and the hypoxia probe using ImageJ software. Data are reported as mean ± SD of three independent experiments. Statistical significance was determined using a two‐tailed Student's *t*‐test (***p < 0.001). Comparison of the cytotoxic effects of **PcNP** and **PcNP@TPZ** against A549 cells with or without pretreatment with **BFG** (60 µm) for 4 h and subsequent light irradiation (λ > 610 nm, 23 mW cm*^−^* ^2^) for 10 min under (c) normoxic (21% O_2_) or (d) hypoxic (2% O_2_) conditions. (e) Cytotoxicity of TPZ under normoxic and hypoxic conditions. For (c–e), data are reported as mean ± SEM of three independent experiments, each performed in quadruplicate. (f) Fluorescence images of calcein‐AM and PI‐stained A549 cells after various treatments. The cells were treated with free TPZ (34 µm), **PcNP**, or **PcNP@TPZ** (both at 5 µm of **Pc–Tz**) for 1 h with or without pre‐treatment with **BFG** (60 µm) for 4 h, followed by light irradiation for 10 min (λ > 610 nm, 23 mW cm^−2^, 13.8 J cm^−2^) or dark treatment. For calcein‐AM: λ_ex_: 488 nm, λ_em_: 500–530 nm; for PI: λ_ex_: 530 nm, λ_em_: 600–620 nm. Scale bar: 100 µm.

We then examined whether bioorthogonal activation of **PcNP@TPZ** could induce intracellular hypoxia following light irradiation. Intracellular hypoxia was visualized using the Image‐iT Red Hypoxia Probe. As shown in Figure 4a (lower row), strong red fluorescence was detected exclusively in cells receiving both **BFG** pretreatment and light irradiation, whereas negligible fluorescence was observed under all other conditions. Quantitative analysis revealed an approximately five‐fold increase in hypoxia‐associated fluorescence intensity (Figure 4b), demonstrating that restoration of the photodynamic activity of **Pc–Tz** resulted in substantial intracellular oxygen consumption. These findings confirm that bioorthogonal activation effectively initiates PDT and establishes a markedly hypoxic intracellular environment suitable for TPZ activation.

Having confirmed efficient intracellular activation in ROS generation and hypoxia, we then investigated whether the released TPZ could enhance the PDT efficacy. A549 cells were incubated with various concentrations of **PcNP** or **PcNP@TPZ** for 1 h with or without pretreatment with **BFG** (60 µm) for 4 h and light irradiation (λ > 610 nm, 23 mW cm^−2^) for 10 min. For comparison, the cells were also treated with various concentrations of free TPZ. All treatments were conducted under a normoxic (21% O_2_) or hypoxic (2% O_2_) condition, and the cell viability was assessed by MTT assay. The results obtained under normoxia are shown in Figure 4c. It can be seen that all treatment groups exhibited negligible dark cytotoxicity, demonstrating the excellent biocompatibility of the nanoassemblies **PcNP** and **PcNP@TPZ**, as well as the bioorthogonal activator. Upon irradiation, **PcNP@TPZ** alone also produced negligible cytotoxicity, indicating that the self‐assembled nanoparticles remained largely quenched in the absence of bioorthogonal activation. In contrast, pretreatment with **BFG** resulted in pronounced photocytotoxicity for both **PcNP** and **PcNP@TPZ**, reflecting efficient restoration of the photosensitizing activity of **Pc–Tz**. Under normoxia, the therapeutic responses of **PcNP** and **PcNP@TPZ** were comparable, suggesting that TPZ remained largely inactive under oxygen‐rich conditions and that the observed cytotoxicity predominantly originated from PDT.

The therapeutic advantage of incorporating TPZ became evident under hypoxia (Figure 4d). For comparison, we first examined the cytotoxicity of TPZ both under normoxic and hypoxic conditions (Figure 4e). When the concentration reached 34 µm, which is roughly the concentration of TPZ in **PcNP@TPZ** at 5 µm of **Pc–Tz**, the cell viability dropped to ca. 80% and 60%, respectively, confirming activation of the prodrug under hypoxia. As shown in Figure 4d, sequential treatment with **BFG** and **PcNP@TPZ** produced slightly greater dark cytotoxicity than free TPZ, suggesting that bioorthogonal disassembly successfully released TPZ and that nanoparticle‐mediated delivery enhanced its intracellular accumulation. Following light irradiation, **PcNP** activated by **BFG** induced substantial photocytotoxicity, although the therapeutic efficacy was slightly lower than that observed under normoxia owing to reduced oxygen availability. Importantly, sequential treatment with **BFG** and **PcNP@TPZ** followed by light irradiation resulted in significantly higher cytotoxicity than all other treatment groups. These results demonstrate that the oxygen depletion caused by PDT effectively promotes TPZ activation, enabling the released prodrug to compensate for the reduced efficiency of oxygen‐dependent PDT. Instead of representing a limitation, PDT‐induced hypoxia therefore becomes an integral component of the therapeutic mechanism.

To visualize this cooperative therapeutic effect directly, treated cells were stained with calcein‐AM and propidium iodide (PI). Consistent with the MTT results, intense calcein fluorescence and minimal PI staining were observed in all dark‐treated groups, confirming negligible cytotoxicity in the absence of photoactivation (Figure 4f). Following irradiation, extensive PI‐positive staining was observed in cells treated with **BFG** and **PcNP** under both normoxic and hypoxic conditions, demonstrating efficient restoration of PDT activity after bioorthogonal activation. Notably, the combination of **BFG** and **PcNP@TPZ** produced the most extensive cell death, particularly under hypoxic conditions, highlighting the complementary actions of restored PDT and hypoxia‐activated chemotherapy. The overall findings establish a chemically programmed therapeutic cascade in which bioorthogonal ligation restores the photodynamic activity of **Pc–Tz**, PDT generates ROS while consuming oxygen, the resulting hypoxia activates TPZ, and the two therapeutic modalities cooperate to produce substantially enhanced anticancer efficacy.

### 2.5 In Vivo Bioorthogonal Activation and Antitumor Therapy

Having demonstrated efficient bioorthogonal activation of these nanoassemblies in cultured cells, we then investigated whether the same activation cascade could be achieved in vivo using HT29 tumor‐bearing nude mice. The in vivo imaging study was performed according to the timeline given in Figure 5a. After tumor inoculation, the mice were intravenously injected with **PcNP** in deionized water (at 30 µm of **Pc–Tz**, 100 µL), followed by intratumoral injection of **BFG** in deionized water (60 µm, 50 µL) after 30 h. Mice that did not receive post‐treatment with **BFG** were used as control. Whole‐body fluorescence imaging was then performed over a further period of 48 h to monitor bioorthogonal activation in real time.

**FIGURE 5:**
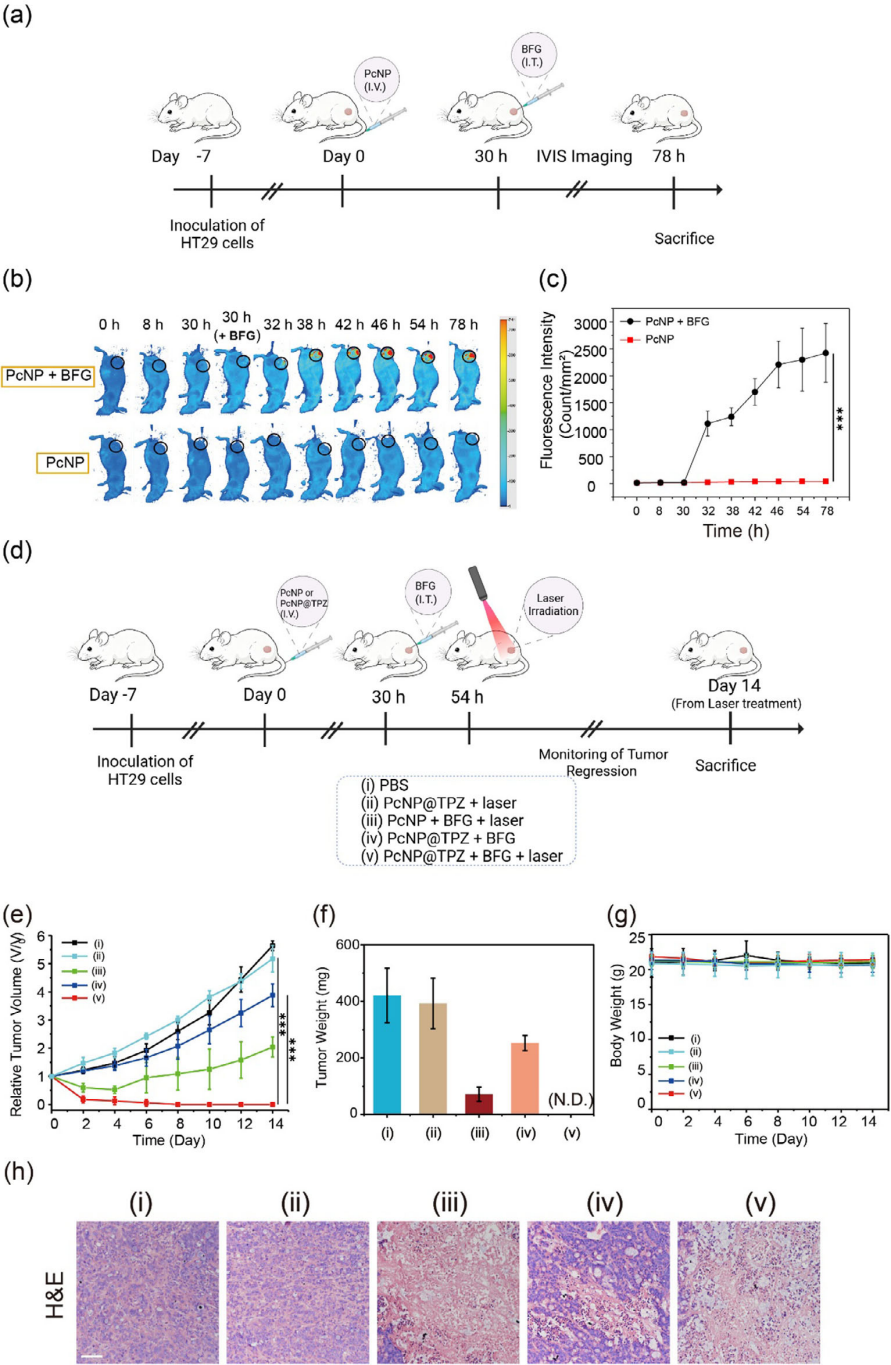
(a) Timeline of the in vivo fluorescence imaging study. (b) Fluorescence images of HT29 tumor‐bearing nude mice after an intravenous injection with **PcNP** in deionized water (at 30 µm of **Pc–Tz**, 100 µL) with or without subsequent intratumoral injection with **BFG** in deionized water (60 µm, 50 µL) after 30 h recorded over a period of 78 h. (c) Quantified fluorescence intensities of the tumors at different time points for these two groups of mice. Data are reported as mean ± SD (*n* = 3). (d) Timeline of the in vivo PDT study. (e) Tumor growth curves for the mice after the following treatments: (i) PBS, (ii) **PcNP@TPZ** + laser, (iii) **PcNP** + **BFG** + laser, (iv) **PcNP@TPZ** + **BFG**, and (v) **PcNP@TPZ** + **BFG** + laser. Drug dose: **PcNP** or **PcNP@TPZ** in deionized water (at 30 µm of **Pc–Tz**, 100 µL); **BFG** in deionized water (60 µm, 50 µL). Light dose: 675 nm, 0.1 W cm^−2^, 10 min. (f) Weights of the tumors in the mice on day 14 after the above treatments. For group (v), the tumors were too small to be measured, and the value is reported as N.D. (not determined). (g) Variation of the body weight of the mice after different treatments. For (e–g), data are reported as mean ± SD (*n* = 4). (h) H&E‐stained images of the tumor slices of the mice after different treatments. Scale bar: 200 µm. Statistical significance was determined using a two‐way repeated measures ANOVA followed by Šídák's multiple comparisons test (***p < 0.001).

This treatment protocol was used to establish whether the bioorthogonal “click‐to‐disassemble” mechanism could operate effectively within the complex in vivo tumor environment and whether such localized activation could produce a therapeutic response. **PcNP** was therefore administered intravenously to permit systemic distribution and tumor accumulation, whereas **BFG** was administered intratumorally to ensure sufficient local availability of the bioorthogonal trigger. It is worth noting that although intratumoral administration bypasses systemic delivery and tumor homing of **BFG**, it does not completely negate the function of GE11, since EGFR‐mediated recognition and cellular uptake can still facilitate interaction of **BFG** with EGFR‐overexpressing tumor cells after local administration.

As shown in Figure 5b, prior to the intratumoral injection of **BFG**, no noticeable fluorescence was detected in the tumors and other regions of the mice, indicating that the self‐assembled **PcNP** remained highly quenched during systemic circulation and tumor accumulation. In marked contrast, intratumoral injection of **BFG** triggered a progressive increase in fluorescence in the tumor region, whereas virtually no fluorescence recovery was observed in mice that did not receive **BFG** (Figure 5b,c). These observations demonstrate that fluorescence activation is governed by the bioorthogonal reaction rather than passive nanoparticle accumulation, thereby confirming that the nanoassemblies remain photodynamically dormant until exposure to the BCN activator.

To further evaluate the spatial selectivity of this activation process, tumors and major organs were harvested 78 h after nanoparticle administration for ex vivo fluorescence imaging. As shown in Figure S13, only weak fluorescence was detected in the heart, liver, spleen, lungs, and kidneys regardless of whether **BFG** was administered. In contrast, tumors receiving **BFG** exhibited a dramatic increase in fluorescence intensity, producing a markedly higher tumor‐to‐background ratio than that observed in untreated mice. These findings confirm that bioorthogonal ligation can selectively activate the self‐quenched nanoassemblies in vivo while minimizing fluorescence activation in normal tissues. Such a high signal‐to‐background ratio is expected to reduce off‐target phototoxicity and improve the precision of photodynamic treatment.

Encouraged by these results, we then evaluated whether bioorthogonal activation could be translated into effective tumor therapy using TPZ‐loaded nanoassemblies (**PcNP@TPZ**). HT29 tumor‐bearing mice were randomly assigned to five treatment groups: (i) PBS, (ii) **PcNP@TPZ** + laser, (iii) **PcNP** + **BFG** + laser, (iv) **PcNP@TPZ** + **BFG**, and (v) **PcNP@TPZ** + **BFG** + laser. **PcNP** or **PcNP@TPZ** was first administered intravenously (at 30 µm of **Pc–Tz**, 100 µL) with or without subsequent intratumoral injection of **BFG** after 30 h. For the light‐treatment groups, tumors were irradiated with a 675 nm diode laser (0.1 W) for 10 min at 54 h post‐injection of nanoparticles, corresponding to the time point at which fluorescence activation had nearly reached its maximum (Figure 5b,c). This treatment schedule (Figure 5d) ensured that PDT was initiated only after efficient bioorthogonal activation of the nanoassemblies had occurred. Tumor regression was then monitored over a period of 14 days from the light treatment.

The tumor growth profiles depicted in Figure 5e clearly demonstrate the contribution of each therapeutic component. Administration of **PcNP@TPZ** together with **BFG** in the absence of laser irradiation (group iv) produced modest inhibition of tumor growth, indicating that TPZ released following bioorthogonal disassembly exerted a measurable chemotherapeutic effect under the intrinsically hypoxic tumor microenvironment. When laser irradiation was applied after treatment with **PcNP** and **BFG** (group iii), substantially greater tumor suppression was achieved owing to restoration of the photodynamic activity of **Pc–Tz** following bioorthogonal activation. Most notably, the combination of **PcNP@TPZ**, **BFG**, and laser irradiation (group v) completely eradicated the tumors around day 8. In contrast, **PcNP@TPZ** followed by laser irradiation in the absence of **BFG** (group ii) exhibited negligible therapeutic efficacy, comparable to that of the PBS control (group i). The tumor weights (Figure 5f) and representative photographs (Figure S14) obtained at the end of treatment were fully consistent with the tumor growth curves. These results clearly demonstrate that passive tumor accumulation alone is insufficient for therapeutic activation; rather, the bioorthogonal reaction is indispensable for restoring the photodynamic activity of **Pc–Tz** and releasing TPZ.

Throughout the 14‐day observation period, no significant changes in body weight were observed in any treatment group (Figure 5g), indicating good systemic tolerability. Histological examination further supported these findings. Hematoxylin and eosin (H&E) staining revealed extensive necrosis and apoptosis in tumors treated with **PcNP** + **BFG** + laser (group iii) or **PcNP@TPZ** + **BFG** + laser (group v), whereas tumors from the remaining groups retained largely intact tissue morphology (Figure 5h). Importantly, no obvious pathological abnormalities were detected in the major organs (Figure S15), suggesting minimal systemic toxicity. Representative photographs of the mice before and during the above treatments are shown in Figure S16, which display a burning mark at the tumor site only for groups iii and v, indicating the occurrence of photodynamic activation. Collectively, these results establish that the present bioorthogonal “click‐to‐disassemble” strategy enables highly selective activation of self‐assembled photosensitizers in vivo and effectively couples PDT with hypoxia‐activated chemotherapy to achieve potent antitumor efficacy while maintaining excellent biosafety.

## 3 Conclusion

In summary, we have developed a bioorthogonal “click‐to‐disassemble” strategy for the site‐specific activation of self‐assembled phthalocyanine nanoassemblies, enabling precise chemo‐photodynamic therapy against hypoxic tumors. The amphiphilic zinc(II) phthalocyanine **Pc–Tz**, comprising three hydrophilic triethylene glycol chains and a hydrophobic tetrazine‐functionalized substituent, spontaneously self‐assembled into photodynamically dormant nanoparticles through aggregation‐induced self‐quenching. Unlike conventional activatable photosensitizers that rely solely on endogenous biochemical stimuli, our strategy exploits an IEDDA reaction to directly modulate the molecular amphiphilicity of the photosensitizer using an exogenous stimulus. Bioorthogonal ligation with a BCN‐bearing activator converted the hydrophobic tetrazine‐containing substituent into a hydrophilic adduct, disrupting the supramolecular packing of the nanoparticles and simultaneously restoring the fluorescence and photodynamic activity of the phthalocyanine.

By integrating an EGFR‐targeting peptide with a cathepsin B‐responsive linker into the BCN activator and co‐encapsulating the hypoxia‐activated prodrug TPZ, we further established a multistage activation cascade that couples receptor‐mediated targeting, intracellular enzymatic cleavage, bioorthogonal nanoparticle disassembly, restoration of photodynamic activity, and hypoxia‐triggered chemotherapy. This chemically programmed sequence was validated through comprehensive solution studies together with cellular and animal experiments, demonstrating highly selective fluorescence activation, efficient ROS generation, controlled TPZ release, and potent antitumor activity with minimal off‐target toxicity. Importantly, the oxygen consumption associated with PDT, traditionally regarded as a limitation, was exploited to enhance TPZ activation, thereby transforming tumor hypoxia into a therapeutic advantage.

To the best of our knowledge, this represents the first example of a self‐assembled photosensitizer nanoassembly whose supramolecular organization and photodynamic function are regulated through bioorthogonal chemistry. More broadly, this work establishes bioorthogonal modulation of molecular amphiphilicity as a general design principle for controlling supramolecular nanomaterials in living systems. We anticipate that this “click‐to‐disassemble” concept will be readily extendable to a wide range of activatable theranostic nanoplatforms, offering new opportunities for the precise spatiotemporal control of imaging, drug release, and combination therapy, and thereby advancing the clinical translation of precision photomedicine.

## Author Contributions

**Jiaxin Shen**: writing – original draft, investigation, methodology, formal analysis, data curation. **Evelyn Y. Xue**: conceptualization, validation, writing – review and editing, formal analysis, supervision. **Caixia Yang**: investigation, methodology. **Peter Chi Keung Cheung**: supervision, resources. **Dennis K. P. Ng**: funding acquisition, validation, writing – review and editing, project administration, resources, supervision.

## Conflicts of Interest

The authors declare no conflicts of interest.

## Supporting information




**Supporting File**: advs78624‐sup‐0001‐SuppMat.pdf.

## Data Availability

The data that support the findings of this study are available from the corresponding authors upon reasonable request.

## References

[1] D. E. J. G. J. Dolmans , D. Fukumura , and R. K. Jain , “Photodynamic Therapy for Cancer,” Nature Reviews Cancer 3, no. 5 (2003): 380–387, 10.1038/nrc1071. 12724736

[2] X. Li , J. F. Lovell , J. Yoon , and X. Chen , “Clinical Development and Potential of Photothermal and Photodynamic Therapies for Cancer,” Nature Reviews Clinical Oncology 17, no. 11 (2020): 657–674, 10.1038/s41571-020-0410-2. 32699309

[3] R. V. Huis in't Veld , J. Heuts , S. Ma , L. J. Cruz , F. A. Ossendorp , and M. J. Jager , “Current Challenges and Opportunities of Photodynamic Therapy Against Cancer,” Pharmaceutics 15, no. 2 (2023): 330, 10.3390/pharmaceutics15020330. PMC9965442 36839652

[4] L. Hong , J. Li , Y. Luo , et al., “Recent Advances in Strategies for Addressing Hypoxia in Tumor Photodynamic Therapy,” Biomolecules 12, no. 1 (2022): 81, 10.3390/biom12010081. PMC8774200 35053229

[5] Y. Peng , C. Shi , Y. Cao , L. Shi , F. Fang , and X. Chen , “Breaking the Hypoxia Barrier: Supramolecular Nanoassemblies Empower Photodynamic Therapy of Tumors,” Angewandte Chemie International Edition 65, no. 26 (2026): 25238, 10.1002/anie.202525238. 42065127

[6] T. C. Pham , V.‐N. Nguyen , Y. Choi , S. Lee , and J. Yoon , “Recent Strategies to Develop Innovative Photosensitizers for Enhanced Photodynamic Therapy,” Chemical Reviews 121, no. 21 (2021): 13454–13619, 10.1021/acs.chemrev.1c00381. 34582186

[7] I. Paramio , T. Torres , and G. de la Torre , “Self‐Assembled Porphyrinoids: One‐Component Nanostructured Photomedicines,” Chem Med Chem 16, no. 16 (2021): 2441–2451, 10.1002/cmdc.202100201. PMC8453889 33900022

[8] Y.‐T. Zhong , Y. Cen , L. Xu , S.‐Y. Li , and H. Cheng , “Recent Progress in Carrier‐Free Nanomedicine for Tumor Phototherapy,” Advanced Healthcare Materials 12, no. 4 (2023): 2202307, 10.1002/adhm.202202307. 36349844

[9] D. Zhang , S. Qin , H. Xu , et al., “Cyanine Nanoassemblies for Synergistic Cancer Therapy: From Aggregate‐State Modulation to Phototheranostic Integration,” Coordination Chemistry Reviews 559 (2026): 217783, 10.1016/j.ccr.2026.217783.

[10] M. Yang , X. Li , and J. Yoon , “Activatable Supramolecular Photosensitizers: Advanced Design Strategies,” Materials Chemistry Frontiers 5, no. 4 (2021): 1683–1693, 10.1039/D0QM00827C.

[11] E. M. Sletten and C. R. Bertozzi , “Bioorthogonal Chemistry: Fishing for Selectivity in a Sea of Functionality,” Angewandte Chemie International Edition 48, no. 38 (2009): 6974–6998, 10.1002/anie.200900942. PMC2864149 19714693

[12] S. Wang , X. Wang , Z. Zhong , et al., “Progress in Bioorthogonal Chemistry for Prodrug Activation and Imaging,” European Journal of Medicinal Chemistry 315 (2026): 118994, 10.1016/j.ejmech.2026.118994. 42184560

[13] B. L. Oliveira , Z. Guo , and G. J. L. Bernardes , “Inverse Electron Demand Diels–Alder Reactions in Chemical Biology,” Chemical Society Reviews 46, no. 16 (2017): 4895–4950, 10.1039/C7CS00184C. 28660957

[14] J. Kim , S. Debnath , E. Kim , et al., “Tetrazine‐Mediated Bioorthogonally Activated Therapeutic (TBAT) Platforms,” Journal of the American Chemical Society 148, no. 9 (2026): 9169–9184, 10.1021/jacs.5c23084. 41771012

[15] K. Porte , B. Renoux , E. Péraudeau , et al., “Controlled Release of a Micelle Payload via Sequential Enzymatic and Bioorthogonal Reactions in Living Systems,” Angewandte Chemie International Edition 58, no. 19 (2019): 6366–6370, 10.1002/anie.201902137. 30856679

[16] J. Song , C. Wu , Y. Zhao , M. Yang , Q. Yao , and Y. Gao , “Bioorthogonal Disassembly of Tetrazine Bearing Supramolecular Assemblies inside Living Cells,” Small 18, no. 2 (2022): 2104772, 10.1002/smll.202104772. 34843166

[17] J. M. Brown , “SR 4233 (Tirapazamine): A New Anticancer Drug Exploiting Hypoxia in Solid Tumours,” British Journal of Cancer 67, no. 6 (1993): 1163–1170, 10.1038/bjc.1993.220. PMC1968495 8512801

[18] I. Genta , E. Chiesa , B. Colzani , T. Moderna , B. Conti , and R. Dorati , “GE11 Peptide as an Active Targeting Agent in Antitumor Therapy: A Minireview,” Pharmaceutics 10 (2018): 2. 10.3390/pharmaceutics10010002 PMC5874815 29271876

[19] M. K. Shim , J. Park , H. Y. Yoon , et al., “Carrier‐Free Nanoparticles of Cathepsin B‐Cleavable Peptide‐Conjugated Doxorubicin Prodrug for Cancer Targeting Therapy,” Journal of Controlled Release 294 (2019): 376–389, 10.1016/j.jconrel.2018.11.032. 30550940

[20] Q. Zhang , X. Wang , J. Chen , et al., “Recent Progress of Porphyrin Metal–Organic Frameworks for Combined Photodynamic Therapy and Hypoxia‐Activated Chemotherapy,” Chemical Communications 60, no. 93 (2024): 13641–13652, 10.1039/D4CC04512B. 39497649

[21] P.‐C. Lo , M. S. Rodríguez‐Morgade , R. K. Pandey , D. K. P. Ng , T. Torres , and F. Dumoulin , “The Unique Features and Promises of Phthalocyanines as Advanced Photosensitisers for Photodynamic Therapy of Cancer,” Chemical Society Reviews 49, no. 4 (2020): 1041–1056, 10.1039/C9CS00129H. 31845688

[22] M. Bai , P.‐C. Lo , J. Ye , C. Wu , W.‐P. Fong , and D. K. P. Ng , “Facile Synthesis of Pegylated Zinc(II) Phthalocyanines via Transesterification and Their In Vitro Photodynamic Activities,” Organic & Biomolecular Chemistry 9, no. 20 (2011): 7028, 10.1039/c1ob05955f. 21858322

[23] S. Kim , M. Fujitsuka , and T. Majima , “Photochemistry of Singlet Oxygen Sensor Green,” The Journal of Physical Chemistry B 117, no. 45 (2013): 13985–13992, 10.1021/jp406638g. 24111566

[24] L. K. B. Tam , L. He , D. K. P. Ng , P. C. K. Cheung , and P.‐C. Lo , “A Tumor‐Targeting Dual‐Stimuli‐Activatable Photodynamic Molecular Beacon for Precise Photodynamic Therapy,” Chemistry – A European Journal 28 (2022): 202201652. 10.1002/chem.202201652 35852020

[25] B. K. Gan , K. Rullah , C. Y. Yong , et al., “Targeted Delivery of 5‐Fluorouracil‐1‐Acetic Acid (5‐FA) to Cancer Cells Overexpressing Epithelial Growth Factor Receptor (EGFR) Using Virus‐Like Nanoparticles,” Scientific Reports 10, no. 1 (2020): 16867, 10.1038/s41598-020-73967-4. PMC7545207 33033330

[26] X. Chen , Z. Zhong , Z. Xu , L. Chen , and Y. Wang , “2′,7′‐Dichlorodihydrofluorescein as a Fluorescent Probe for Reactive Oxygen Species Measurement: Forty Years of Application and Controversy,” Free Radical Research 44, no. 6 (2010): 587–604, 10.3109/10715761003709802. 20370560

