# Supplementary material for: Bioorthogonal Click‐to‐Disassemble Phthalocyanine Nanoassemblies for Precise Chemo‐Photodynamic Therapy against Hypoxic Tumors
Source: Adv Sci (Weinh). 2026 Nov 9:e78624. Online ahead of print. doi: 10.1002/advs.78624 (PMC13749000; doi:10.1002/advs.78624)
Supplement: Supplementary file 1 [file ADVS-9999-e78624-s001.pdf]

<u>**Supporting Information**</u>

Bioorthogonal Click-to-Disassemble Phthalocyanine Nanoassemblies for Precise Chemo-Photodynamic Therapy Against Hypoxic Tumors

Jiaxin Shen[1] | Evelyn Y. Xue[1,2] | Caixia Yang[1] | Peter Chi Keung Cheung[3] | Dennis K. P. Ng[1]

[1]Department of Chemistry, The Chinese University of Hong Kong, Shatin, N.T., Hong Kong, China | [2]Department of Biomedical Engineering, The Chinese University of Hong Kong, Shatin, N.T., Hong Kong, China | [3]School of Life Sciences, The Chinese University of Hong Kong, Shatin, N.T., Hong Kong, China

Correspondence: Evelyn Y. Xue (yangxue@cuhk.edu.hk) | Dennis K. P. Ng (dkpn@cuhk.edu.hk)

Contents

Experimental Section

SCHEME S1 Synthesis of amphiphilic **Pc–Tz**.

SCHEME S2 Synthesis of **BF**, **BG**, and **BFG**.

FIGURE S1 (a) Change in the relative hydrodynamic diameter of **PcNP** in dark at room temperature over a period of 6 days. (b) TEM image of **PcNP**.

FIGURE S2 MALDI-TOF mass spectrum of FRRG.

FIGURE S3 MALDI-TOF mass spectrum of GE11.

FIGURE S4 MALDI-TOF mass spectrum of FRRG-GE11.

FIGURE S5 (a) HPLC chromatogram of **BF**. (b) ESI mass spectrum of the fraction collected at 22 min.

FIGURE S6 (a) HPLC chromatogram of **BG**. (b) ESI mass spectrum of the fraction collected at 23 min.

FIGURE S7 (a) HPLC chromatogram of **BFG**. (b) ESI mass spectrum of the fraction collected at 23 min.

FIGURE S8 Change in fluorescence spectrum of **PcNP** (at 5 μM of **Pc–Tz**) in deionized water in the (a) absence and presence of (b) **BF**, (c) **BG**, and (d) **BFG** (all at 120 μM) over a period of 35 min.

FIGURE S9 MALDI-TOF mass spectrum of the reaction mixture after treating **PcNP** (at 5 μM of **Pc–Tz**) in deionized water with **BF** (120 μM) at room temperature for 35 min.

FIGURE S10 Change in fluorescence spectrum of SOSG (10 μM) in deionized water containing **PcNP** (at 5 μM of **Pc–Tz**) (a) in the absence of a BCN derivative or after the treatment with (b) **BF**, (c) **BG**, or (d) **BFG** (all at 120 μM) for 35 min, followed by light irradiation (λ > 610 nm) over a period of 16 min. The fluorescence spectra were recorded at 1-min intervals.

FIGURE S11 (a) Confocal microscopic images of A549 cells after sequential incubation with **BFG** (120 μM, 4 h) and **PcNP** (at 5 μM of **Pc–Tz**, 1 h) with or without pre-treatment with CA-074Me (25 μM, 2 h). As a non-activated control, cells were incubated with **PcNP** (at 5 μM of **Pc–Tz**) only. The fluorescence of ZnPc is indicated in red, while cell nuclei were counterstained with DAPI (in blue). Scale bar: 20 μm. (b) Quantification of the mean intracellular fluorescence intensities for the three groups.

FIGURE S12 (a) Confocal microscopic images of A549 cells after incubation with **PcNP** (at 5 μM of **Pc–Tz**) for 1 h with or without pre-incubation with different concentrations of **BG** (0–120 μM) for 4 h. Scale bar: 20 μm. (b) Intracellular fluorescence intensities under the above conditions determined by flow cytometry. Data are reported as mean ± SD of three independent experiments. Cytotoxicity of **PcNP** (at 5 μM of **Pc–Tz**) triggered by **BG** (120 μM) against (c) A549, (d) A431, (e) HT29, and (f) HepG2 cells, both in the dark and upon light irradiation (λ > 610 nm, 23 mW cm^{-2}) for 10 min.

FIGURE S13 (a) Representative ex vivo images and (b) quantified fluorescence intensities of the tumors and some major organs harvested at 78 h post-injection of **PcNP** for the two groups of mice (n = 3).

FIGURE S14 Representative photographs of the tumors in the mice on Day 14 after different treatments.

FIGURE S15 Representative H&E-stained images of the major organs after different treatments.

FIGURE S16 Representative photographs of the mice during the course of different treatments.

FIGURE S17 ^{1}H NMR spectrum of **3** in $CDCl_3$ with a trace amount of pyridine-d_5.

FIGURE S18 High-resolution ESI mass spectrum of **3**. The experimental (left) and simulated (right) isotopic patterns of the molecular ion signal are shown in the inset.

FIGURE S19 ^{1}H NMR spectrum of **Pc–Tz** in DMSO-d_6.

FIGURE S20 High-resolution ESI mass spectrum of **Pc–Tz**. The experimental (top) and simulated (bottom) isotopic patterns of the protonated and sodiated molecular ion signals are shown in the lower left and right, respectively.

Experimental Section

General

N,*N*-Dimethylformamide (DMF) and tetrahydrofuran (THF) were purified using an Inert solvent purification system. All other solvents and reagents were of reagent grade and used without further purification. All reactions except peptide synthesis were conducted under a nitrogen atmosphere and monitored by thin-layer chromatography (TLC) on Merck silica gel 60F254 plates. Chromatographic purification was performed using a silica gel column (Macherey-Nagel, 230-400 mesh). Size-exclusion chromatography was carried out on Bio-Rad Bio-Beads S-X1 beads (200-400 mesh) using THF as the eluent. Compounds **1** [R1], **2** [R2], **4** [R3], and **5** [R4] were prepared as described.

^{1}H NMR spectra were acquired on a Bruker Avance III 400 MHz NMR spectrometer in $CDCl_3$ or DMSO-d_6. Spectra were referenced internally by using the residual solvent [δ = 7.26 ppm (for $CDCl_3$), δ = 2.50 ppm (for DMSO-d_6)] resonance relative to $SiMe_4$. Electrospray ionization (ESI) mass spectra were recorded on a Thermo Finnigan MAT 95 XL mass spectrometer. Matrix-assisted laser desorption/ionization time-of-flight (MALDI-TOF) mass spectra were recorded on a Bruker Autoflex LRF mass spectrometer. Electronic absorption and steady-state fluorescence spectra were recorded on a Shimadzu UV-1800 UV-Vis spectrophotometer and a Horiba FluoroMax-4 spectrofluorometer, respectively.

Preparation of Phthalocyanine 3

A mixture of phthalonitrile **1** (1.00 g, 3.45 mmol), phthalonitrile **2** (0.20 g, 0.72 mmol), and anhydrous $Zn(OAc)_2$ (0.66 g, 3.60 mmol) in n-pentanol (70 mL) was heated to 80 °C. Afterward,

1,8-diazabicyclo[5.4.0]undec-7-ene (DBU) (1.0 mL, 6.69 mmol) was added. The mixture was then stirred at 140 ℃ for 6 h. After cooling, the mixture was poured into 10 mL of petroleum ether, and the collected green solid was purified by column chromatography on silica gel with CH_2Cl_2/CH_3OH (10:1 v/v) as the eluent, yielding ZnPc **3** as a green solid (0.12 g, 14%). ^1H NMR (400 MHz, $CDCl_3$ with a trace amount of pyridine-d_5): δ 8.16-8.24 (m, 2 H, Ar-H), 7.34-7.45 (m, 2 H, Ar-H), 4.70 (br. s, 6 H, OCH_2), 4.34-4.39 (m, 2 H, OCH_2), 4.14-4.25 (m, 6 H, OCH_2), 3.91-3.99 (m, 6 H, OCH_2), 3.82-3.87 (m, 6 H, OCH_2), 3.73-3.78 (m, 6 H, OCH_2), 3.60-3.65 (m, 6 H, OCH_2), 3.41 (s, 9 H, OCH_3), 1.78-1.85 (m, 2 H, CH_2), 1.37-1.52 (m, 4 H, CH_2), 0.90-0.98 (m, 3 H, CH_3). The signals of the phthalocyanine ring protons were too broad to be detected due to aggregation. HRMS (ESI): *m/z* calcd for $C_{65}H_{72}N_8O_{15}Zn$ $[M]^+$ 1268.4414, found 1268.4378.

Preparation of Phthalocyanine Pc–Tz

A mixture of **3** (100 mg, 79 μmol) and NaOH (100 mg, 2.5 mmol) in CH_3OH/H_2O/THF (5:1:1 v/v/v, 8 mL) was stirred at room temperature for 30 min. The mixture was then acidified with 5 M HCl to a pH of 2–3. The resulting green precipitate was collected through centrifugation (ca. 100 mg), which was then treated directly with tetrazine-substituted phenol **4** (31 mg, 0.16 mmol) in the presence of 4-(dimethylamino)pyridine (DMAP) (30 mg, 0.25 mmol) and 1-ethyl-3-(3-dimethylaminopropyl)carbodiimide (EDC) (18 mg, 0.12 mmol) in THF (5 mL). The mixture was stirred at room temperature for 2 h. The volatiles were then evaporated under reduced pressure. The residue was purified by column chromatography on silica gel with $CHCl_3/CH_3OH$ (30:1 v/v) as the eluent, yielding **Pc–Tz** as a green solid (78 mg, 72%). ^1H

NMR (400 MHz, DMSO-d_6): δ 8.69-9.33 (several br. s, 8 H, Pc-H_α), 8.58 (dd, *J* = 3.2, 8.8 Hz, 2 H, Ar-H), 8.32-8.39 (m, 2 H, Ar-H), 7.90-8.02 (m, 2 H, Ar-H), 7.57-7.73 (m, 6 H, Pc-H_β and Ar-H), 4.61-4.71 (m, 6 H, OCH_2), 4.03-4.10 (m, 6 H, OCH_2), 3.78-3.83 (m, 6 H, OCH_2), 3.68-3.72 (m, 6 H, OCH_2), 3.61-3.65 (m, 6 H, OCH_2), 3.49-3.52 (m, 6 H, OCH_2), 3.25 (s, 9 H, OCH_3), 3.02 (s, 3 H, CH_3). HRMS (ESI): *m/z* calcd for $C_{69}H_{69}N_{12}O_{15}Zn$ $[M+H]^+$ 1369.4291, found 1369.4285.

Preparation of PcNP

A solution of **Pc–Tz** (6.8 μg, 5 nmol) in DMSO (20 μL) was injected into deionized water (980 μL) under vigorous magnetic stirring at room temperature. The resulting nanoparticle suspension was transferred into a dialysis tube (MWCO 1500 Da) and dialyzed against deionized water for 12 h at room temperature to remove the residual DMSO. The dialysis medium was refreshed every 4 h to ensure complete removal of the residual DMSO.

Preparation of PcNP@TPZ

Pc–Tz (6.8 μg, 5 nmol) and TPZ (22 μg, 125 nmol) were dissolved in DMSO (20 μL), and the mixture was injected into deionized water (980 μL) under vigorous magnetic stirring at room temperature. The resulting nanoparticle suspension was transferred into a dialysis tube (MWCO 1500 Da) and dialyzed against deionized water for 12 h at room temperature to remove free TPZ and residual DMSO. The dialysis medium was refreshed every 4 h to ensure complete removal of these substances.

Determination of Encapsulation Efficiency

PcNP@TPZ was disrupted using 1% (v/v) Triton X-100 to completely release the encapsulated TPZ. The absorbance at 462 nm was recorded using a UV–Vis spectrophotometer, and the concentration of TPZ was calculated using an established standard calibration curve. Encapsulation efficiency (EE) (%) was calculated using the following equation:

$$EE\ (\%) = (W_{encapsulated}/W_{initial}) \times 100$$

where = $W_{initial}$ is the amount of TPZ initially added, $W_{encapsulated}$ is the amount of TPZ quantified after nanoparticle disruption. For the above conditions, the concentration of TPZ encapsulated/released was found to be 34 μM, giving an EE value of 27%.

General Procedure of Peptide Synthesis and Purification

The linear peptide resins with different sequences were synthesized manually using a standard 9-fluorenylmethoxycarbonyl (Fmoc) solid-phase peptide synthesis protocol with commercially available *N*-α-Fmoc-protected amino acids as starting materials. Rink amide resin was used as the solid support. A 20% piperidine solution in DMF was used to remove the Fmoc protecting group, while 1-[bis(dimethylamino)methylene]-1*H*-1,2,3-triazolo[4,5-*b*]pyridinium 3-oxide (HATU) was utilized to activate the carboxyl group. The swelled resin was treated with piperidine (20%) in DMF for 20 min twice before being coupled with the first amino acid. An excess of the Fmoc-protected amino acids (4 equiv.), HATU (4 equiv.), and *N,N*-diisopropylethylamine (DIPEA) (8 equiv.) were employed for each coupling step in DMF at room temperature. After the final coupling, the resin was treated with a solution of 95% trifluoroacetic acid (TFA), 2.5% triisopropylsilane (TIPS), and 2.5% H_2O for 1 h to detach the

peptides from the resin and remove the protecting groups. The resin was then filtered out, and the filtrate was precipitated with diethyl ether. After centrifugation, the supernatant was discarded, and the crude peptides were purified by reverse-phase high-performance liquid chromatography (HPLC).

Reverse-phase HPLC separation was performed on an XBridge BEH300 C18 column (5 μm, 4.6 mm × 150 mm) at a flow rate of 1 mL min^{-1} for analytical purpose or on a XBridge BEH300 Prep C18 column (5 μm, 10 mm × 250 mm) at 3 mL min^{-1} for preparative purpose. The system included a Waters 1525 binary pump and a Waters 2998 photodiode array detector. HPLC solvents were of HPLC grade, with the following conditions: solvent A = 0.1% TFA in acetonitrile; solvent B = 0.1% TFA in deionized water. The gradient was: 5% A + 95% B for 5 min, changed to 15% A + 85% B in 10 min, and then changed to 100% A in 30 min, maintained under this condition for 5 min, changed to 0% A + 100% B in 5 min, and then maintained under this condition for an additional 5 min.

Synthesis of Peptides FRRG, GE11, and FRRG-GE11

Peptides FRRG, GE11 (YHWYGYTPQNVI), and FRRG-GE11 were synthesized as described above. To check the purity, a portion of the peptide resin was treated with a solution (1 mL) containing a mixture of TFA (95%), TIPS (2.5%), and H_2O (2.5%) for 2 h to detach it from the resin and remove the protecting groups. The resin was removed by filtration, and the filtrate was precipitated by the addition of diethyl ether. After centrifugation, the supernatant was removed. The yellow solid obtained was redissolved in DMF (0.3 mL) and then precipitated again by the addition of diethyl ether. This purification procedure was repeated twice, and the

product was dried in vacuo before HPLC analysis. The crude peptides were purified by reverse-phase HPLC. The purity of all the peptide was found to be >95% by HPLC analysis. For FRRG: MS (MALDI-TOF): *m/z* calcd for $C_{23}H_{38}N_{10}O_5$ $[M]^+$ 534.303, found 534.553. For GE11: MS (MALDI-TOF): *m/z* calcd for $C_{75}H_{97}N_{17}O_{19}$ $[M+Na]^+$ 1563.685, found 1563.972. For FRRG-GE11: MS (MALDI-TOF): *m/z* calcd for $C_{98}H_{133}N_{27}O_{23}$ $[M]^+$ 2056.007, found 2056.005.

Synthesis of BF

A mixture of FRRG peptide (10 mg, 18.4 μmol), 4-nitrophenyl carbonate-modified BCN **5** (7 mg, 22 μmol), and DIPEA (40 μL) in DMF (1.5 mL) was stirred at room temperature for 6 h. After evaporation, the residue was analyzed by liquid chromatography – mass spectrometry (LC–MS) on a XSelect CSH C18 column (5 μm, 4.6 mm × 250 mm) at a flow rate of 0.8 mL min^{-1} using a Waters system equipped with a Waters Quaternary Solvent Manager-R, a Waters 2998 photodiode array detector, and a Waters single quadrupole detector 2. The solvents used were of LCMS grade. The conditions used for analysis were set as follows: solvent A = 0.01% formic acid in acetonitrile; solvent B = 0.01% formic acid in deionized water. The gradient was: 100% A + 0% B in the first 5 min, changed to 0% A + 100% B in 30 min, maintained under this condition for 10 min, and then changed to 100% A + 0% B in 15 min. The conjugate was obtained as a white solid (5 mg, 38%) with > 95% purity. MS (ESI): *m/z* calcd for $C_{34}H_{52}N_{10}O_7$ $[M+2H]^{2+}$ 356.20, found 356.25.

Synthesis of BG

A mixture of GE11 peptide (8 mg, 5 μmol), 4-nitrophenyl carbonate-modified BCN **5** (5 mg,

16 μmol), and DIPEA (20 μL) in DMF (1 mL) was stirred at room temperature for 6 h. After evaporation, the residue was analyzed by LC–MS as described above, yielding the conjugate as a white solid (5 mg, 56%) with >95% purity. MS (ESI): *m*/*z* calcd for $C_{86}H_{111}N_{17}O_{21}$ $[M+2H]^{2+}$ 858.90, found 858.73.

Synthesis of BFG

A mixture of FRRG-GE11 peptide (11 mg, 5 μmol), 4-nitrophenyl carbonate-modified BCN **5** (2 mg, 6.3 μmol), and DIPEA (20 μL) in DMF (1 mL) was stirred at room temperature for 6 h. After evaporation, the residue was analyzed by LC–MS as described above, yielding the conjugate as a white solid (6 mg, 54%) with >95% purity. MS (ESI): *m*/*z* calcd for $C_{109}H_{150}N_{27}O_{25}$ $[M+5H]^{5+}$ 447.42, found 447.44.

Study of Singlet Oxygen Generation

PcNP (at 5 μM of **Pc–Tz**) in deionized water was treated with **BF**, **BG**, or **BFG** (all at 120 μM) for 35 min. Subsequently, Singlet Oxygen Sensor Green (SOSG, 10 μM) was added, and then the mixtures were exposed to red light from a 300 W halogen lamp, which had passed through a water tank for cooling and a color filter with a cut-on wavelength at 610 nm. The mixtures were irradiated for 16 min ($\lambda > 610$ nm, 23 mW cm^{-2}, 22.1 J cm^{-2}). The fluorescence spectrum of SOSG was recorded in 1-min interval with excitation at 504 nm. **PcNP** without the treatment with these activators was used for comparison.

Release of TPZ

The release of TPZ from **PcNP@TPZ** was investigated using a dialysis method. **PcNP@TPZ** (at 5 μM of **Pc–Tz**) in deionized water was treated with **BF** (120 μM), and the mixture was gently stirred at room temperature. The resulting solution was immediately transferred into a dialysis bag with a molecular weight cut-off (MWCO) of 3.5 kDa, which was immersed in deionized water as the release medium. Aliquots of the external medium were collected at 5 min intervals over a period of 35 min for analysis. The release of TPZ was quantified by measuring the absorbance at 462 nm using a UV-Vis spectrophotometer. Control experiments were performed under identical conditions without the addition of **BF**.

Cell Lines and Culture Conditions

HT29 human colorectal adenocarcinoma cells (ATCC, no. HTB-38) were maintained in Roswell Park Memorial Institute (RPMI) 1640 medium (Invitrogen, no. 23400-021) supplemented with 10% fetal bovine serum (FBS) (ThermoFisher Scientific, cat. no. 10270-106) and penicillin-streptomycin solution (100 units mL^{-1} and 100 μg mL^{-1}, respectively). A549 human lung carcinoma cells (ATCC® CCL-185™), A431 human epidermoid carcinoma cells (ATCC® CRL-1555™), and HepG2 human hepatocellular carcinoma cells (ATCC® HB-8065™) were maintained in Dulbecco's modified Eagle medium (DMEM) (ThermoFisher Scientific, cat. no. 12100-046) supplemented with 10% FBS and penicillin-streptomycin solution (100 units mL^{-1} and 100 μg mL^{-1}). All the cells were grown at 37 °C in a humidified 5% CO_2 atmosphere.

Confocal Microscopic Study of In Vitro Fluorescence Activation

Approximately 2 × 10^5 cells in the corresponding medium (2 mL) were seeded on a confocal dish and incubated overnight at 37 °C in a humidified 5% CO_2 atmosphere. After being rinsed with PBS, A549 cells were incubated with **BFG** or **BG** (15, 30, 60, and 120 μM) at 37 °C for 4 h and then in the medium with or without the presence of **PcNP** (at 5 μM of **Pc–Tz**) at 37 °C for 1 h. The cells were then rinsed with PBS twice. A431, HT29, and HepG2 cells were pre-treated with **BFG** (120 μM) or in neat culture medium for 4 h and then incubated with **PcNP** (at 5 μM of **Pc–Tz**) for 1 h. After being rinsed with PBS, these cells were examined using a Leica TCS SP8 confocal microscope equipped with a 638 nm laser. **Pc–Tz** was excited at 638 nm, and its fluorescence was monitored at 650–750 nm. The images were digitized and analyzed using Leica Application Suite X software.

Flow Cytometric Analysis of In Vitro Fluorescence Activation

After the treatments mentioned above, the cells were harvested by 0.25% trypsin-ethylenediaminetetraacetic acid (Invitrogen, 0.1 mL) for 5 min. The activity of trypsin was quenched with a serum-containing medium (0.5 mL), and the mixture was centrifuged at 500 rpm for 5 min. The pellet was washed with PBS and suspended in PBS (1.0 mL). The intracellular fluorescence intensities were then analyzed using a BD FACSVerse flow cytometer with 10^4 cells counted in each sample. **Pc–Tz** was excited by an argon laser at 640 nm, and the emitted fluorescence was monitored in the range of 650–800 nm. Data were analyzed using BD FAC-Suite software, and all experiments were performed in triplicate.

Cathepsin B Inhibition Study

Approximately 2 × 10^5 A549 cells in DMEM (2 mL) were seeded on a confocal dish and incubated overnight at 37 °C in a humidified 5% CO_2 atmosphere. Three groups were examined. For the baseline control group, the cells were rinsed with PBS and incubated with **PcNP** (at 5 μM of **Pc–Tz**) at 37 °C for 1 h without any trigger. For the activation group, the cells were rinsed with PBS, incubated with **BFG** (120 μM) at 37 °C for 4 h, and then with **PcNP** (at 5 μM of **Pc–Tz**) at 37 °C for 1 h. For the inhibition group, the cells were pre-treated with CA-074Me (25 μM) in culture medium at 37 °C for 2 h, then incubated with **BFG** (120 μM) at 37 °C for 4 h and subsequently with **PcNP** (at 5 μM of **Pc–Tz**) at 37 °C for 1 h, with CA-074Me (25 μM) being maintained in the medium throughout the pre-treatment and **BFG** incubation periods. After the treatments, the cells were rinsed with PBS twice, and the nuclei were counterstained with 4',6-diamidino-2-phenylindole (DAPI) (1 μg mL^{-1}) at 37 °C for 10 min, followed by two further washes with PBS. The cells were then examined using a Leica STELLARIS confocal microscope equipped with a 638 nm laser. **Pc–Tz** was excited at 638 nm, and its fluorescence was monitored at 650–750 nm. The images were digitized and analyzed using Leica Application Suite X software.

Bioorthogonal Activation in Photocytotoxicity of PcNP

Cytotoxicity of **PcNP** against A549, A431, HT29, and HepG2 cells was evaluated using the MTT assay. Approximately 1 × 10^4 cells were seeded in 96-well plates and incubated overnight at 37 °C in a humidified atmosphere containing 5% CO_2. The cells were incubated with a serum-free culture medium containing **BFG** or **BG** at varying concentrations (0, 15, 30, 60, and 120 μM) for 4 h. After being rinsed with PBS, the cells were incubated with **PcNP** (at 5

μM of **Pc–Tz**) for 1 h. They were then rinsed with PBS and refed with culture medium before being irradiated for 10 min (λ > 610 nm, 23 mW cm^{-2}, 13.8 J cm^{-2}). Afterward, 50 μL of an MTT solution (3 mg mL^{-1}) was added to each well, and the cells were incubated at 37 °C for 4 h. Subsequently, the MTT solution was removed, and the insoluble formazan crystals were dissolved in 100 μL DMSO. The absorbance at 490 nm due to the formazan product was measured using a microplate reader (Tecan Spark 10M).

Study of Intracellular ROS Generation

Intracellular ROS generation was assessed using 2',7'-dichlorodihydrofluorescein diacetate (H_2DCFDA) with confocal microscopy. Approximately 2 × 10^5 A549 cells were incubated in the culture medium with or without the presence of **BFG** (60 μM) for 4 h. Following this, the cells were washed with PBS and incubated with **PcNP** (at 5 μM of **Pc–Tz**) for 1 h. The cells were then stained with H_2DCFDA (10 μM) at 37 °C for 30 min. After being rinsed with PBS twice and refed with a serum-free medium (1 mL), the cells were irradiated with light for 3 min (λ > 610 nm, 23 mW cm^{-2}, 4.1 J cm^{-2}) or left in the dark before being examined with a Leica TCS SP8 high-speed confocal microscope equipped with a 638 nm laser. The fluorescence of DCF in the cells was monitored at 515–580 nm upon excitation at 488 nm. Images were digitized and analyzed using Leica Application Suite X software. For qualitative analysis, the images were processed with ImageJ software.

Detection of Intracellular Hypoxia

A549 cells (2 × 10^4 per well) were incubated in culture medium with or without **BFG** (60 μM)

for 4 h. After incubation, the cells were washed with PBS and then incubated with **PcNP@TPZ** (at 5 μM of **Pc–Tz**) for 1 h. The cells were irradiated for 3 min (λ > 610 nm, 23 mW cm^{-2}, 4.1 J cm^{-2}) or kept in the dark. After rinsing with PBS twice, the cells were stained with Image-iT™ Red Hypoxia Reagent (15 μM) for 5 min. The fluorescence of the hypoxia probe was imaged using a Leica TCS SP8 confocal microscope, with excitation at 488 nm and monitoring at 580–620 nm. The images were digitized and analyzed using Leica Application Suite X software. For qualitative analysis, the images were processed with ImageJ software.

Study of Combined Cytotoxicity

Cytotoxicity of free TPZ, **PcNP**, and **PcNP@TPZ** against A549, A431, HT29, and HepG2 cells was evaluated using the MTT assay. Approximately 1 × 10^4 cells were seeded in 96-well plates and incubated overnight at 37 °C in a humidified atmosphere containing 5% CO_2. To explore the combined cell-killing efficiency of **PcNP@TPZ** under normoxic or hypoxic conditions, the cells were transferred to an incubator with either 21% or 2% oxygen for 24 h. The cells were treated with various concentrations of free TPZ, **PcNP**, or **PcNP@TPZ** for 1 h with or without pre-treatment with **BFG** (60 μM) for 4 h. The cells were rinsed with PBS and refed with culture medium, followed by light irradiation for 10 min (λ > 610 nm, 23 mW cm^{-2}, 13.8 J cm^{-2}) or dark treatment. After incubation in either a 21% or 2% oxygen atmosphere for 12 h, the cell viability was determined using the MTT assay.

Study of Cytotoxicity by Calcein-AM and Propidium Iodide Staining

Approximately 2 × 10^5 A549 cells in the culture medium (2 mL) were seeded on a confocal

dish and incubated overnight at 37 °C in a humidified 5% CO_2 atmosphere. To visualize the cell-killing effect of drugs under normoxic or hypoxic conditions, the cells were transferred to an incubator with either 21% or 2% oxygen for 24 h. The cells were then treated with free TPZ (34 μM), **PcNP**, or **PcNP@TPZ** (both at 5 μM of **Pc–Tz**) for 1 h with or without pre-treatment with **BFG** (60 μM) for 4 h. The cells were rinsed with PBS and refed with fresh culture medium, followed by light irradiation for 10 min (λ > 610 nm, 23 mW cm^{-2}, 13.8 J cm^{-2}) or dark treatment. The cells were stained with calcein-AM (2 μM) and propidium iodide (PI) (4.5 μM) at 37 ºC for 20 min in a serum-free medium. The fluorescence images of the cells were recorded by a Leica TCS SP8 confocal microscope. Calcein-AM and PI were excited at 488 and 530 nm, and the emission was collected between 500–530 nm and 600–620 nm, respectively. The images were digitized and analyzed using Leica Application Suite X software.

In Vivo Biodistribution

Female Balb/c nude mice (20-25 g) were obtained from the Laboratory Animal Services Centre of The Chinese University of Hong Kong. All animal experiments were approved by the Animal Experimentation Ethics Committee of the University (Ref. No. 21-347-GRF). The mice were kept under pathogen-free conditions with free access to food and water. HT29 cells (about 5×10^5 cells in 200 μL PBS) were inoculated subcutaneously on the back of the mice. When the tumors reached approximately 100 mm^3, the mice were intravenously injected with **PcNP** in deionized water (at 30 μM of **Pc–Tz**, 100 μL), followed by intratumoral injection of **BFG** in deionized water (60 μM, 50 μL) after 30 h. In vivo fluorescence image was captured before and after injection at various time points up to 78 h using an Odyssey infrared imaging

system (excitation wavelength = 680 nm, emission wavelength ≥ 700 nm). The images were digitized and analyzed by using Odyssey imaging system software (9201-500). After the in vivo imaging studies, the mice were euthanized at 78 h post-injection of **PcNP**. The tumors and major organs were harvested for fluorescence intensity measurements. Three mice were used for each experiment.

In Vivo Antitumor Efficacy and H&E Staining

HT29 cells (approximately 5×10^5 cells in 200 μL PBS) were inoculated subcutaneously on the back of the mice. Tumor volume was calculated using the formula: Tumor Volume = Length × Width^2 / 2. Once the tumors reached approximately 100 mm^3, the HT29 tumor-bearing mice were divided into five groups: (i) PBS, (ii) **PcNP@TPZ** + laser, (iii) **PcNP** + **BFG** + laser, (iv) **PcNP@TPZ** + **BFG**, and (v) **PcNP@TPZ** + **BFG** + laser. Mice received intravenous injections of PBS (100 μL), **PcNP**, or **PcNP@TPZ** (both at 30 μM of **Pc–Tz**, 100 μL). For the **BFG** treatment groups, **BFG** (60 μM, 50 μL) was administered intratumorally 30 h after the injection of **PcNP@TPZ**. At 54 h post-injection of the nanoparticles, the tumors were irradiated with a diode laser (Biolitec Ceralas) at 675 nm, operating at 0.1 W. Illumination was applied for 10 min (total fluence = 60 J cm^{-2}). Tumor size was monitored periodically for 14 days. In each group, two mice were retained and euthanized one day after laser irradiation. The tumors were harvested for H&E staining, and internal organs were harvested after 14 days treatment. Tissues were fixed with 10% neutral buffered formalin, dehydrated with ethanol, and embedded in paraffin for sectioning and H&E staining, followed by examination using optical microscopy.

Statistical Analysis

All quantitative data are presented as mean ± SD or SEM, as specified in the corresponding figure legends, from at least three independent replicates. Statistical comparisons between two groups were performed using a two-tailed Student's t-test. For time-course measurements involving repeated measurements on the same animals, statistical significance was analyzed by a two-way repeated measures ANOVA followed by Šídák's multiple comparisons test. Differences were considered statistically significant at $p < 0.05$. Significance levels are denoted in the figures as *$p < 0.05$, **$p < 0.01$, and ***$p < 0.001$; "n.s." indicates no significant difference.

References

1. G. T. Ducharme, Z. LaCasse, T. Sheth, I. V. Nesterova, and E. E. Nesterov, "Design of Turn-On Near-Infrared Fluorescent Probes for Highly Sensitive and Selective Monitoring of Biopolymers," *Angewandte Chemie International Edition* 59 (2020): 8440–8444.

2. F. Li, Q. Liu, Z. Liang, J. Wang, M. Pang, W. Huang, W. Wu, and Z. Hong, "Synthesis and Biological Evaluation of Peptide-Conjugated Phthalocyanine Photosensitizers with Highly Hydrophilic Modifications," *Organic and Biomolecular Chemistry* 14 (2016): 3409–3422.

3. J. Yang, M. R. Karver, W. Li, S. Sahu, and N. K. Devaraj, "Metal-Catalyzed One-Pot Synthesis of Tetrazines Directly from Aliphatic Nitriles and Hydrazine," *Angewandte Chemie International Edition* 51 (2012): 5222–5225.

4. T. Rady, M. Mosser, M. Nothisen, S. Erb, I. Dovgan, S. Cianférani, A. Wagner, and G. Chaubet, "Bicyclo[6.1.0]nonyne Carboxylic Acid for the Production of Stable Molecular Probes," *RSC Advances* 11 (2021): 36777–36780.

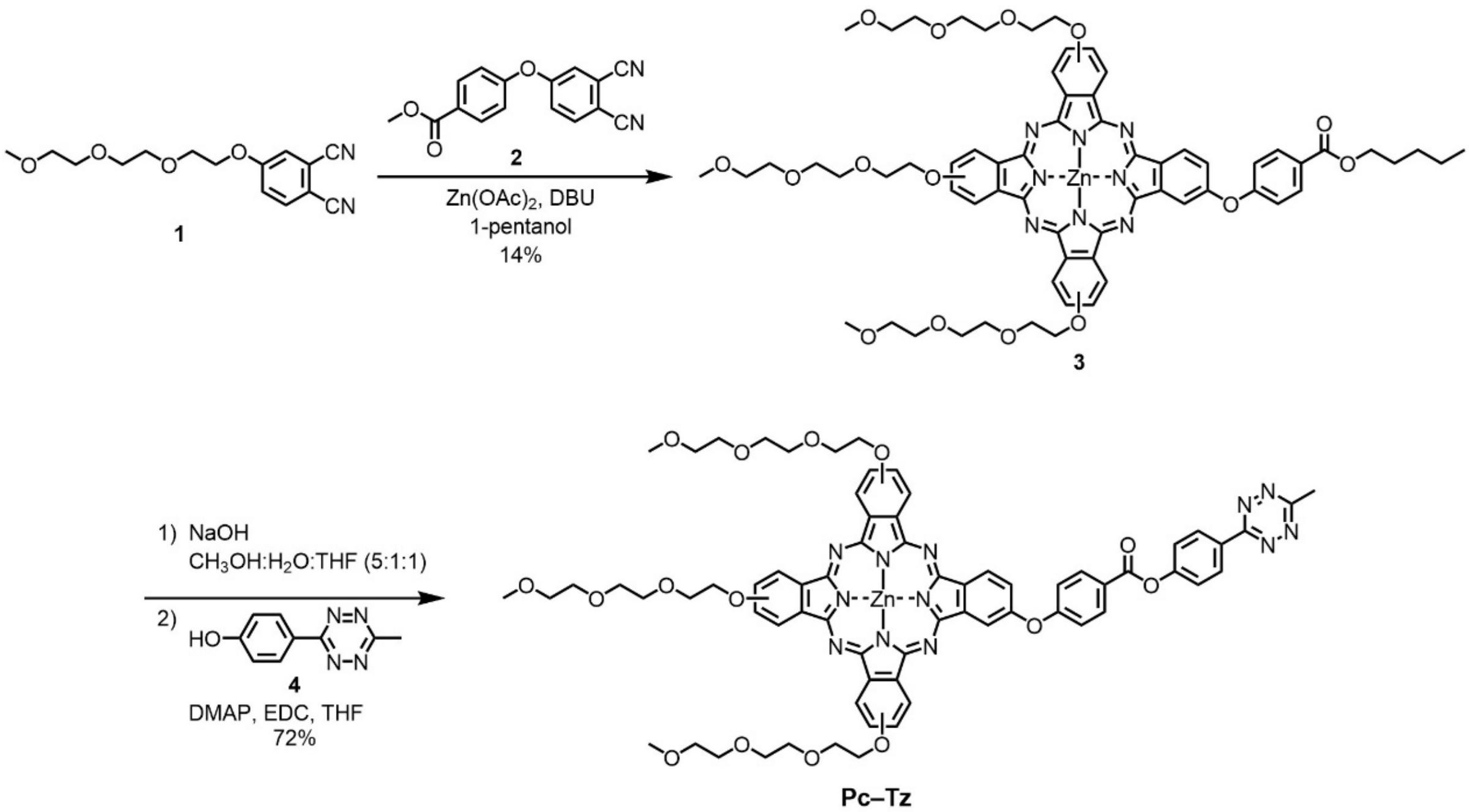

SCHEME S1 | Synthesis of amphiphilic **Pc–Tz**.

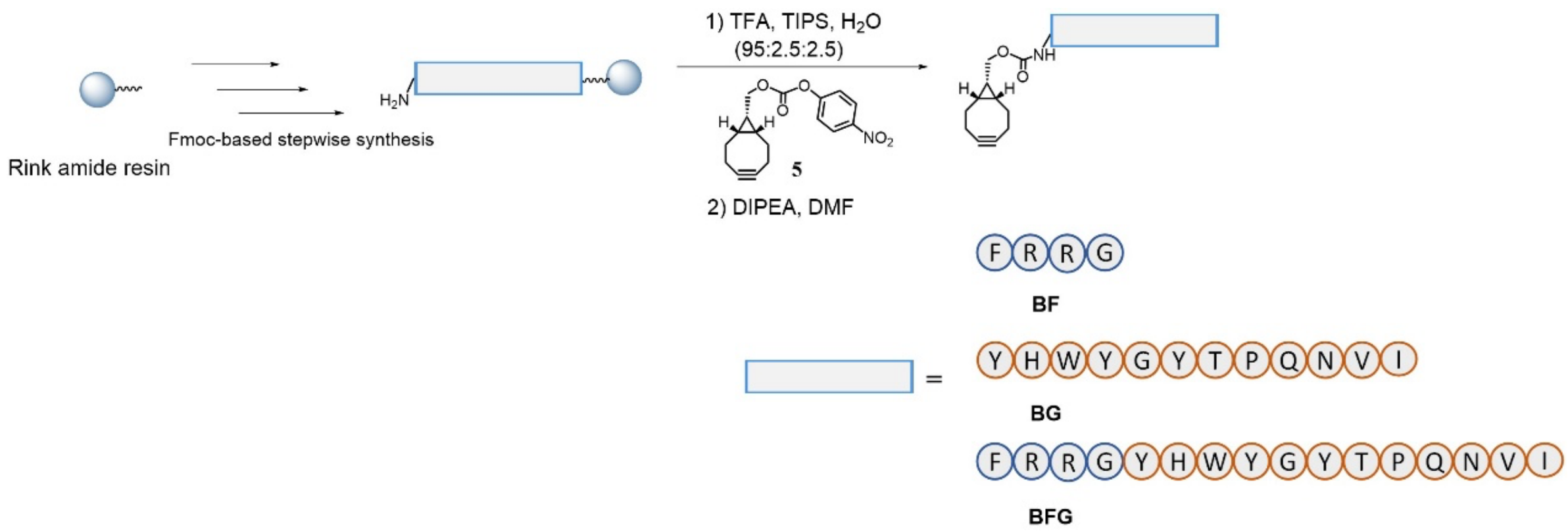

SCHEME S2 | Synthesis of **BF**, **BG**, and **BFG**.

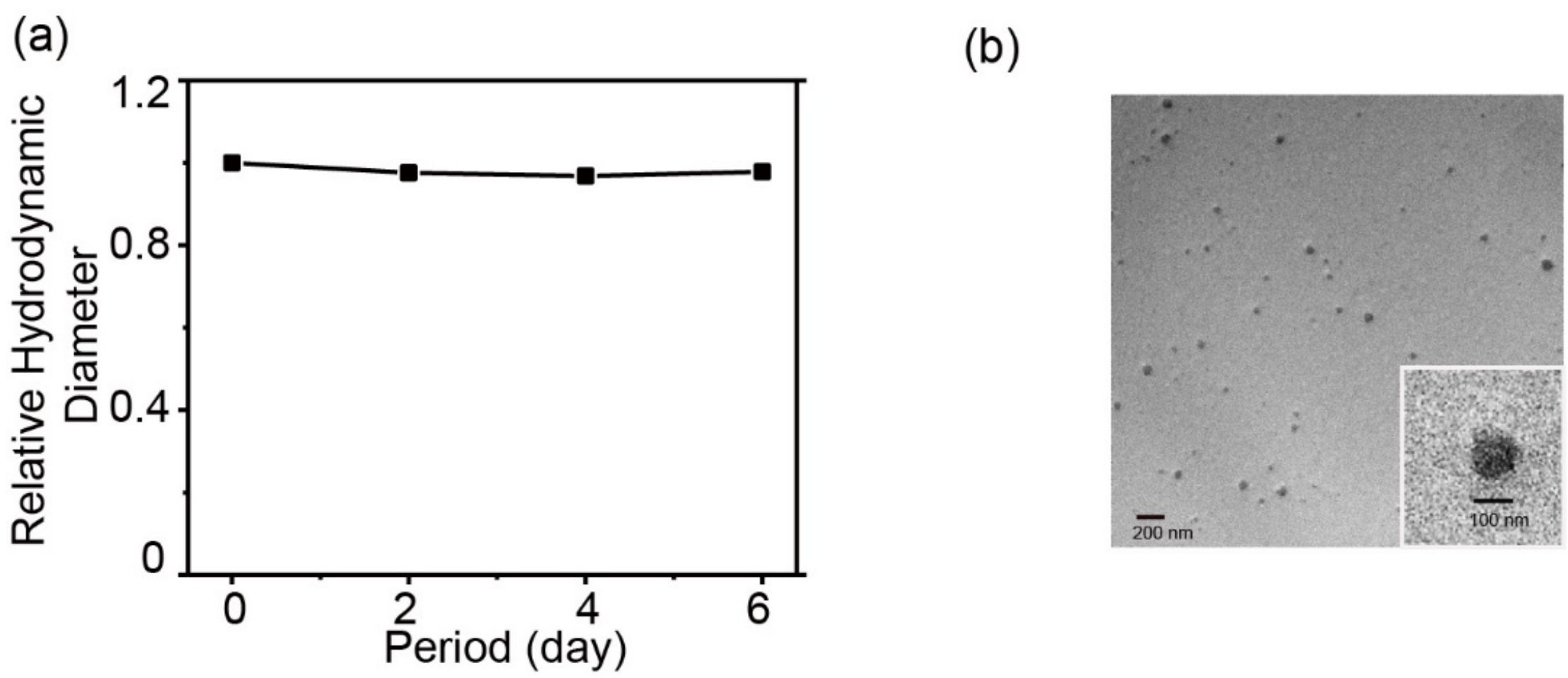

FIGURE S1 | (a) Change in the relative hydrodynamic diameter of **PcNP** in dark at room temperature over a period of 6 days. (b) TEM image of **PcNP**.

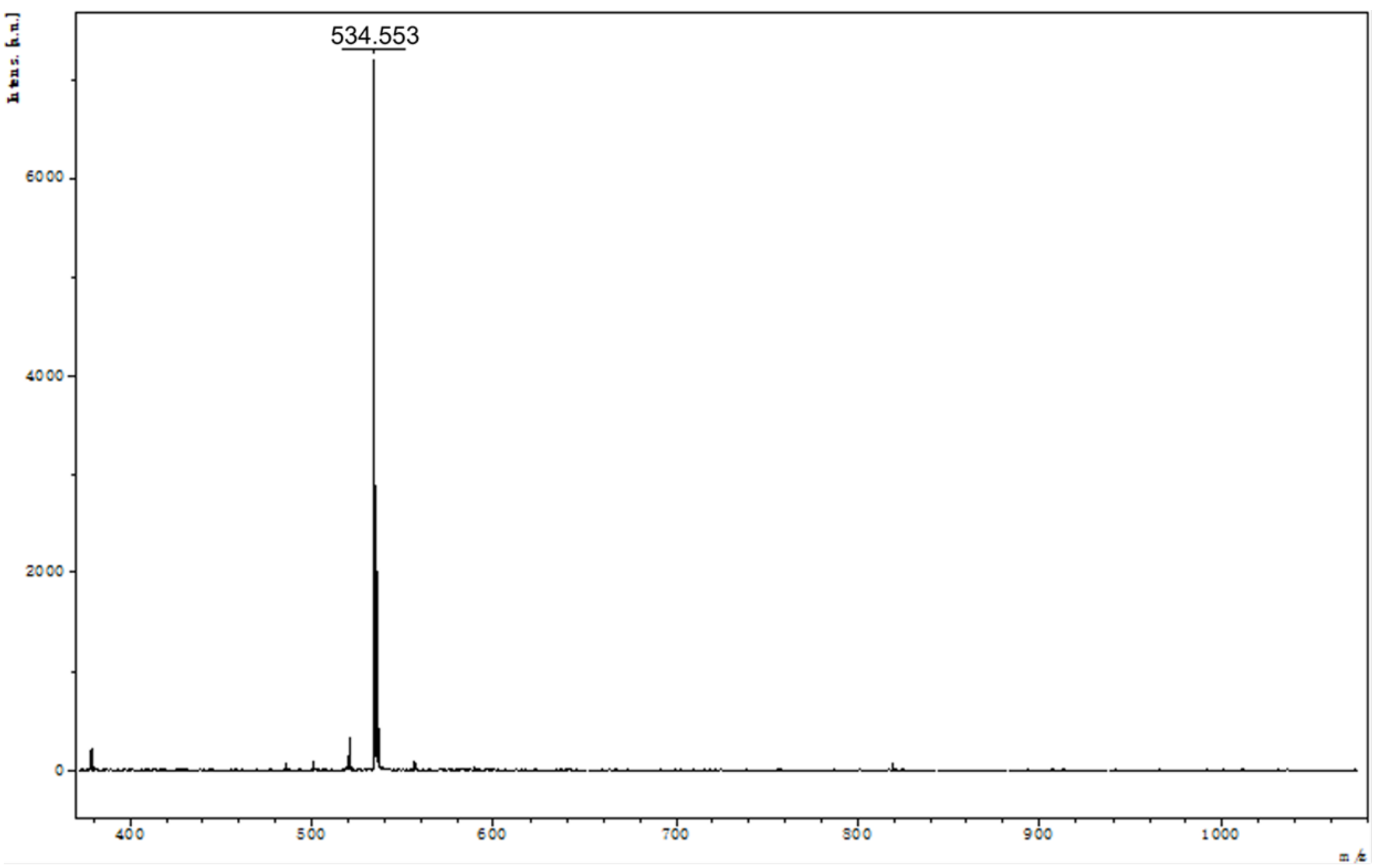

FIGURE S2 | MALDI-TOF mass spectrum of FRRG.

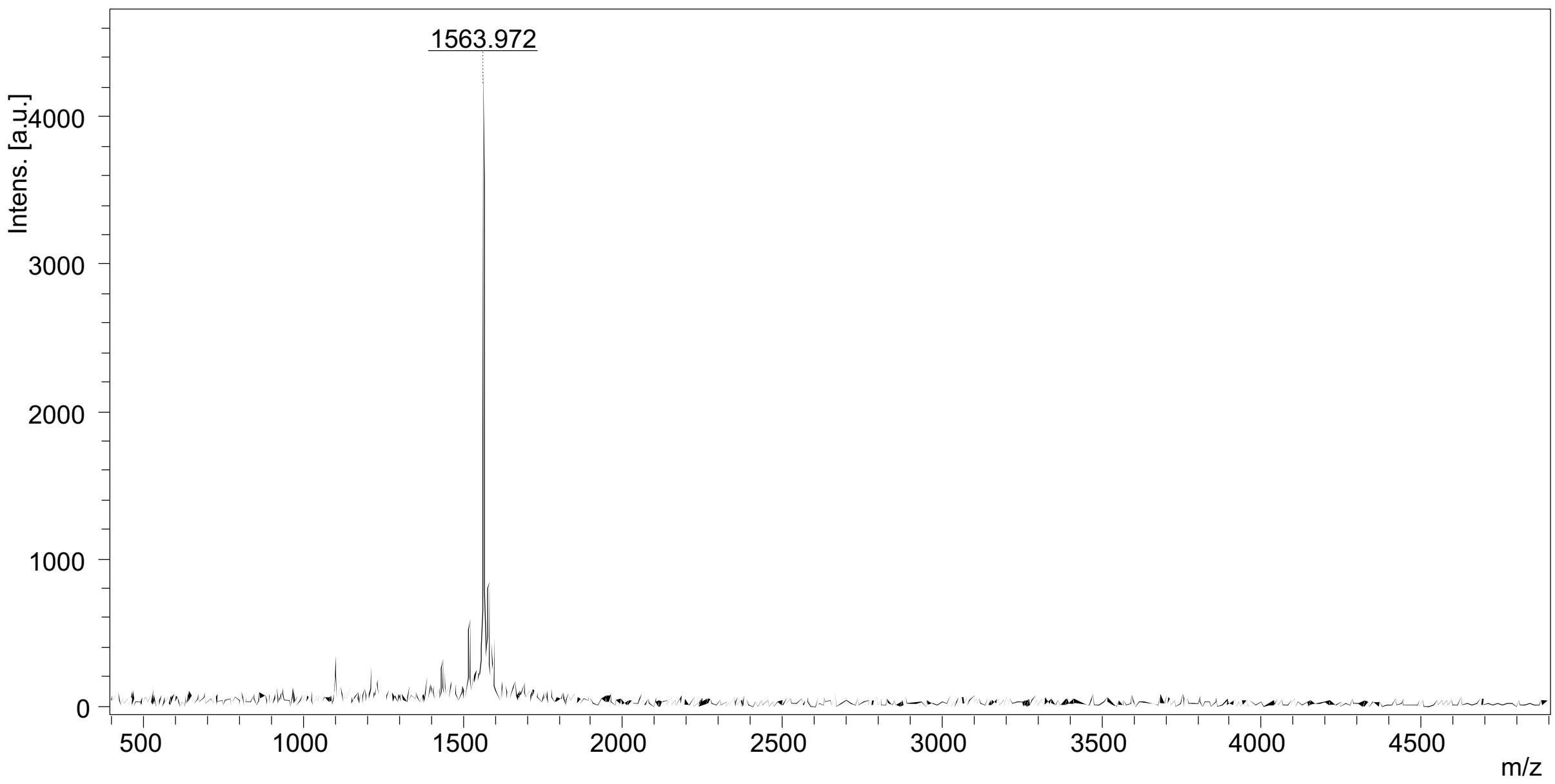

FIGURE S3 | MALDI-TOF mass spectrum of GE11.

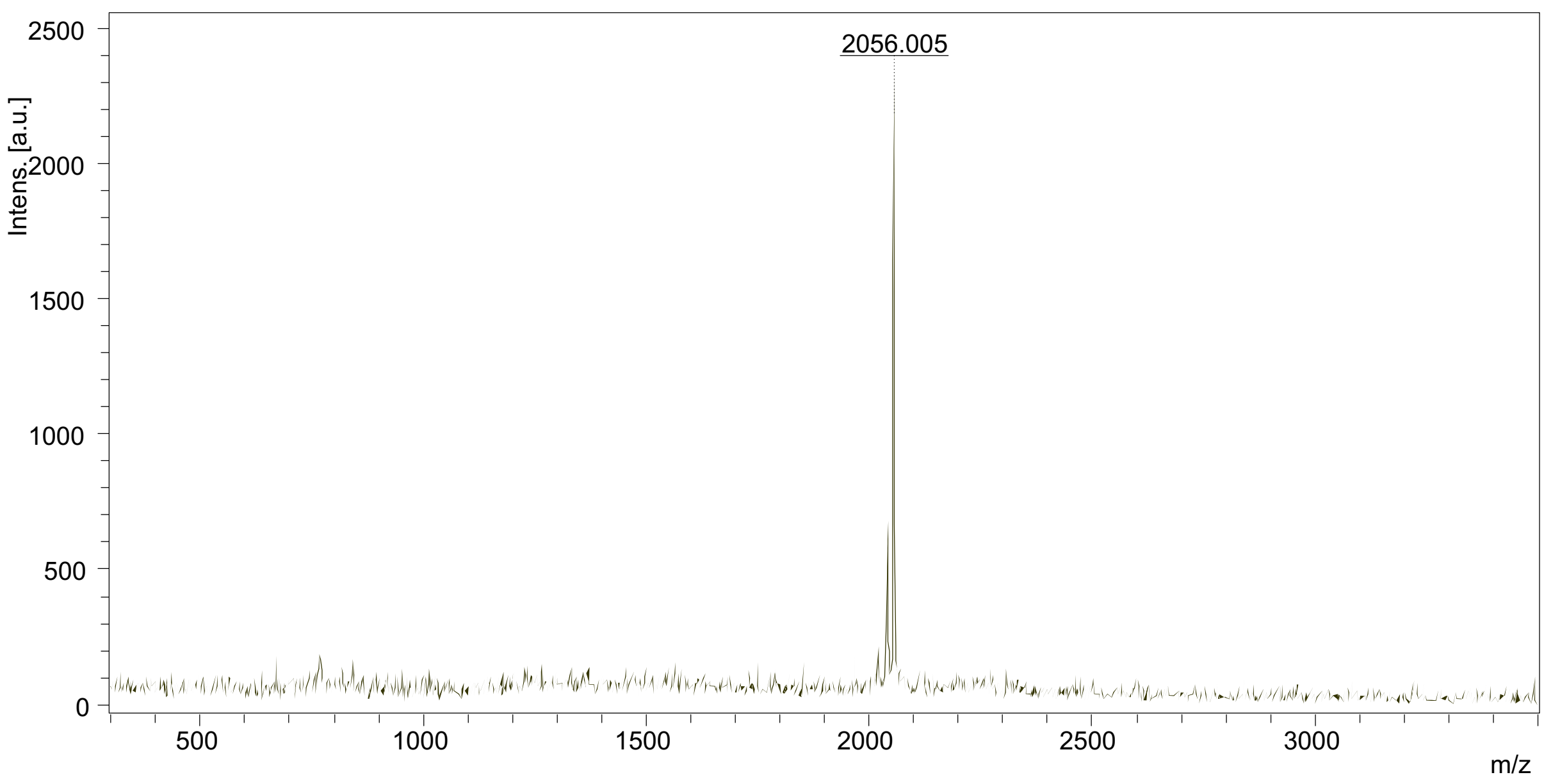

FIGURE S4 | MALDI-TOF mass spectrum of FRRG-GE11.

(a)

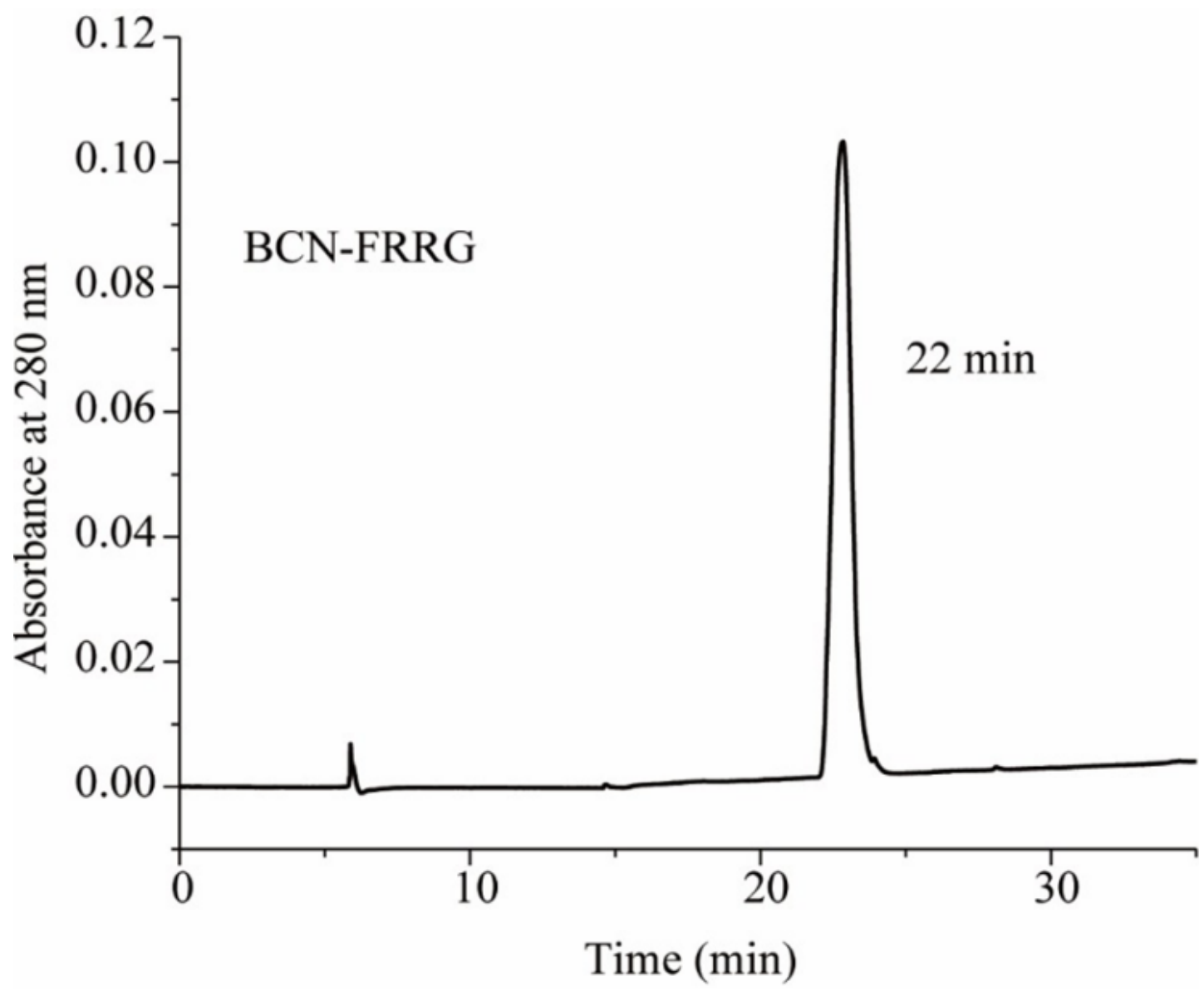

(b)

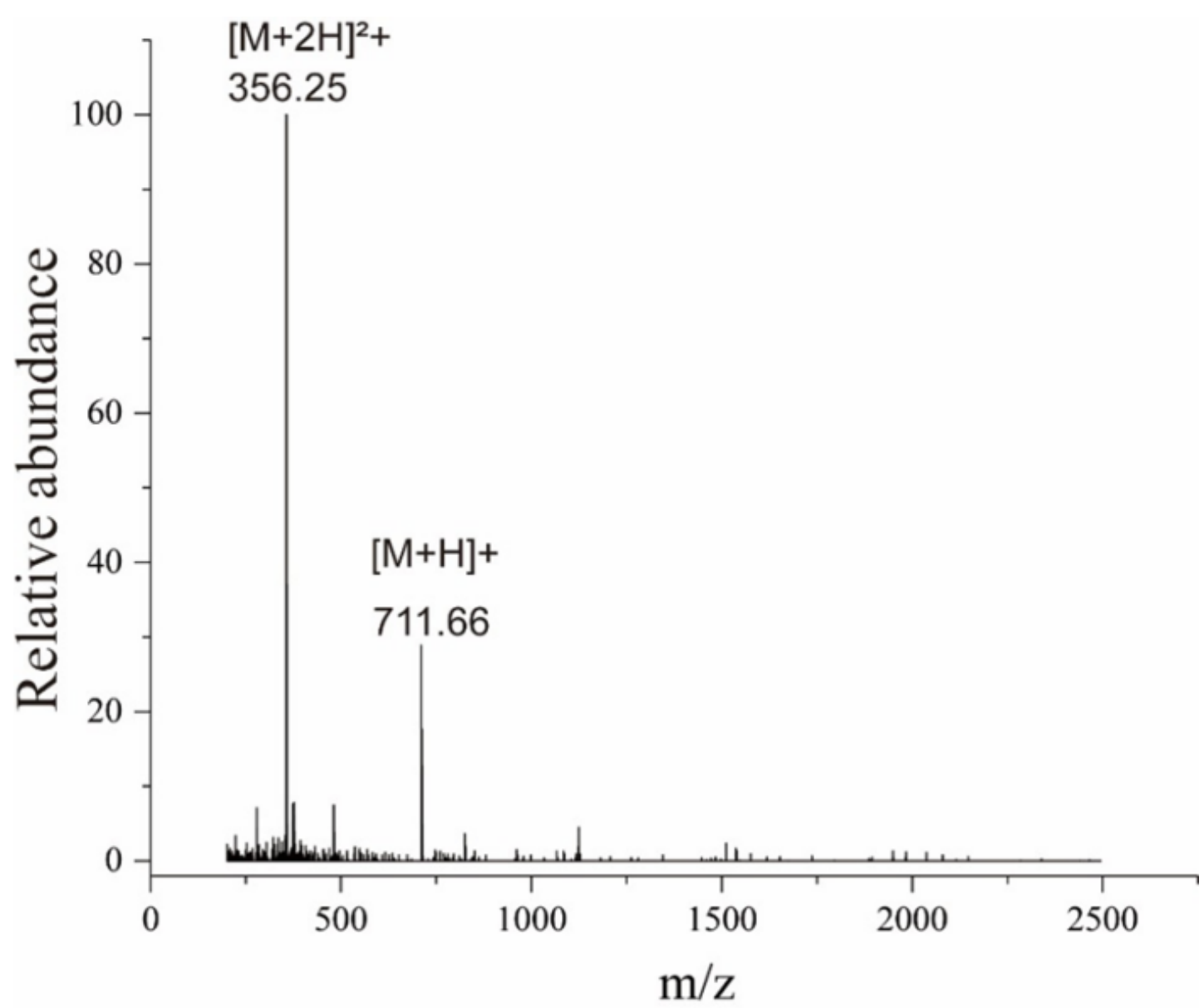

FIGURE S5 | (a) HPLC chromatogram of **BF**. (b) ESI mass spectrum of the fraction collected at 22 min.

(a)

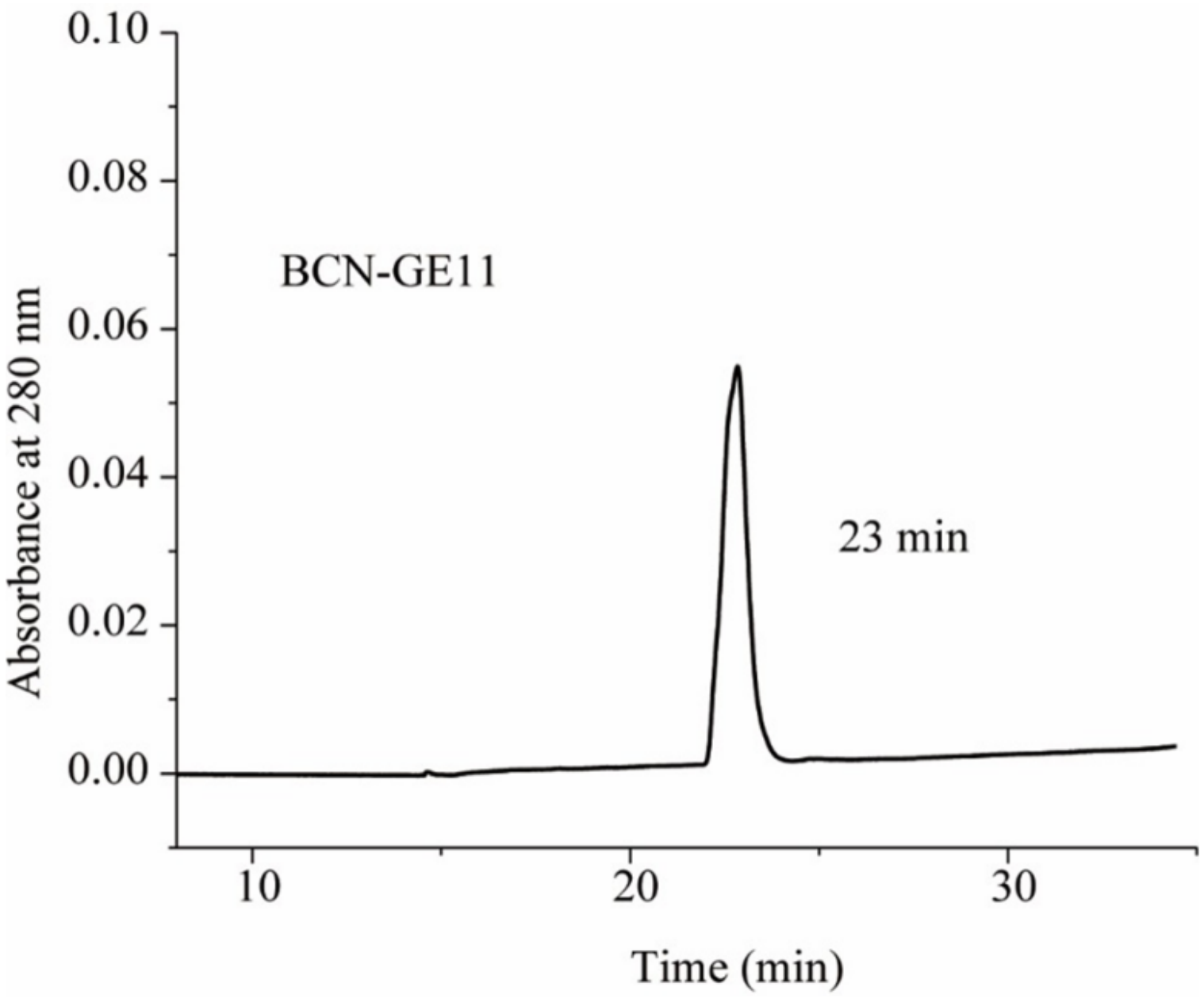

(b)

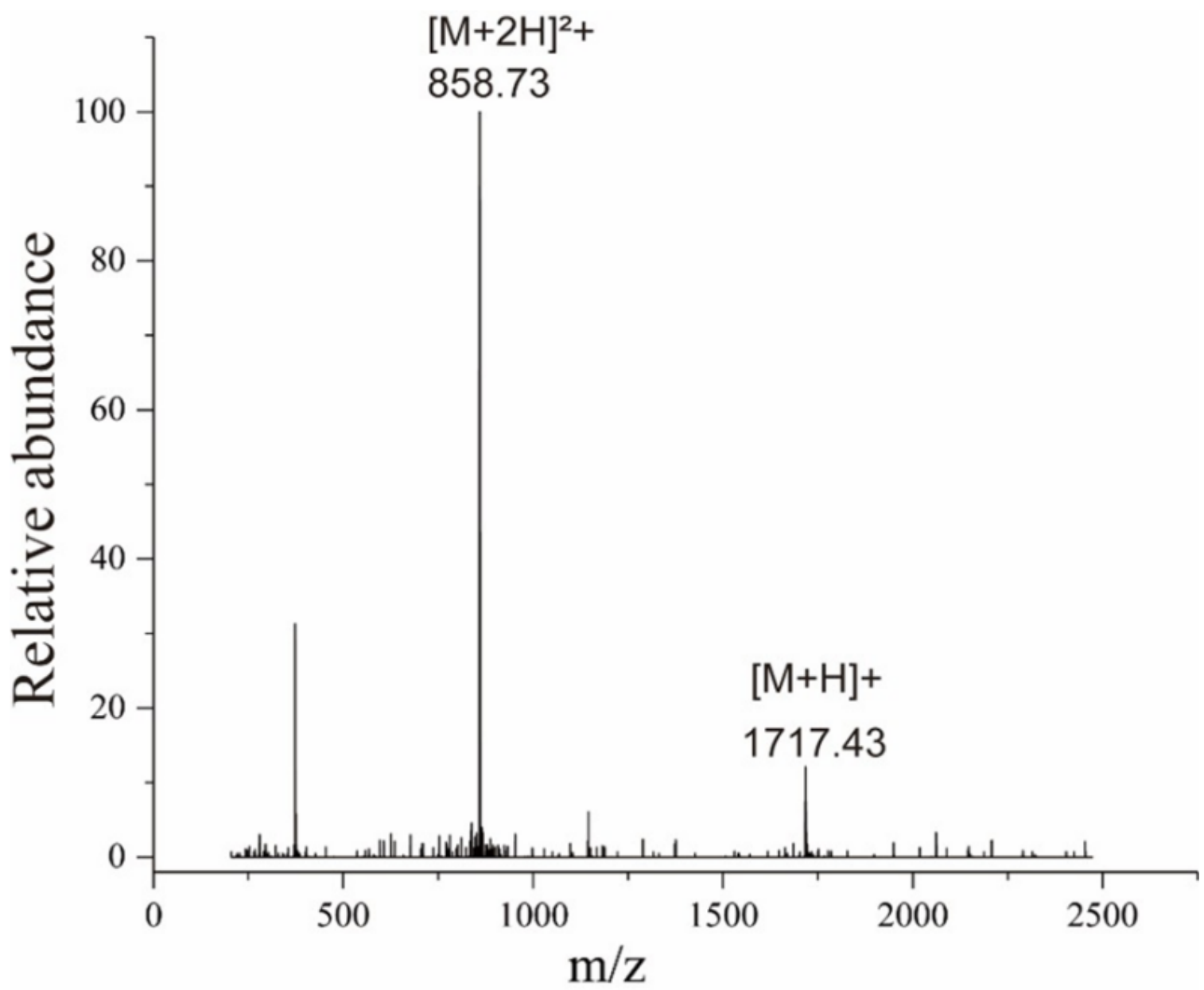

FIGURE S6 | (a) HPLC chromatogram of **BG**. (b) ESI mass spectrum of the fraction collected at 23 min.

(a)

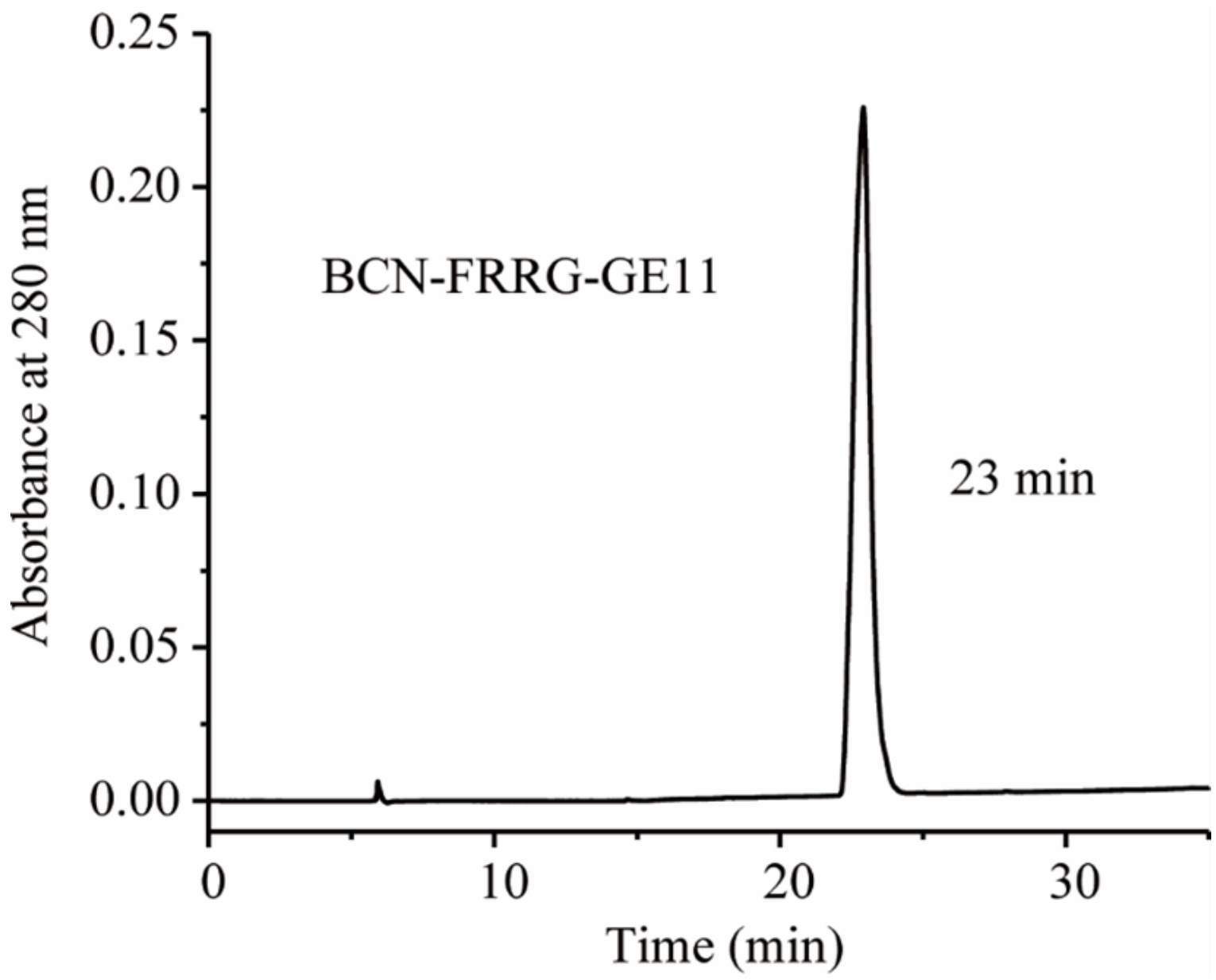

(b)

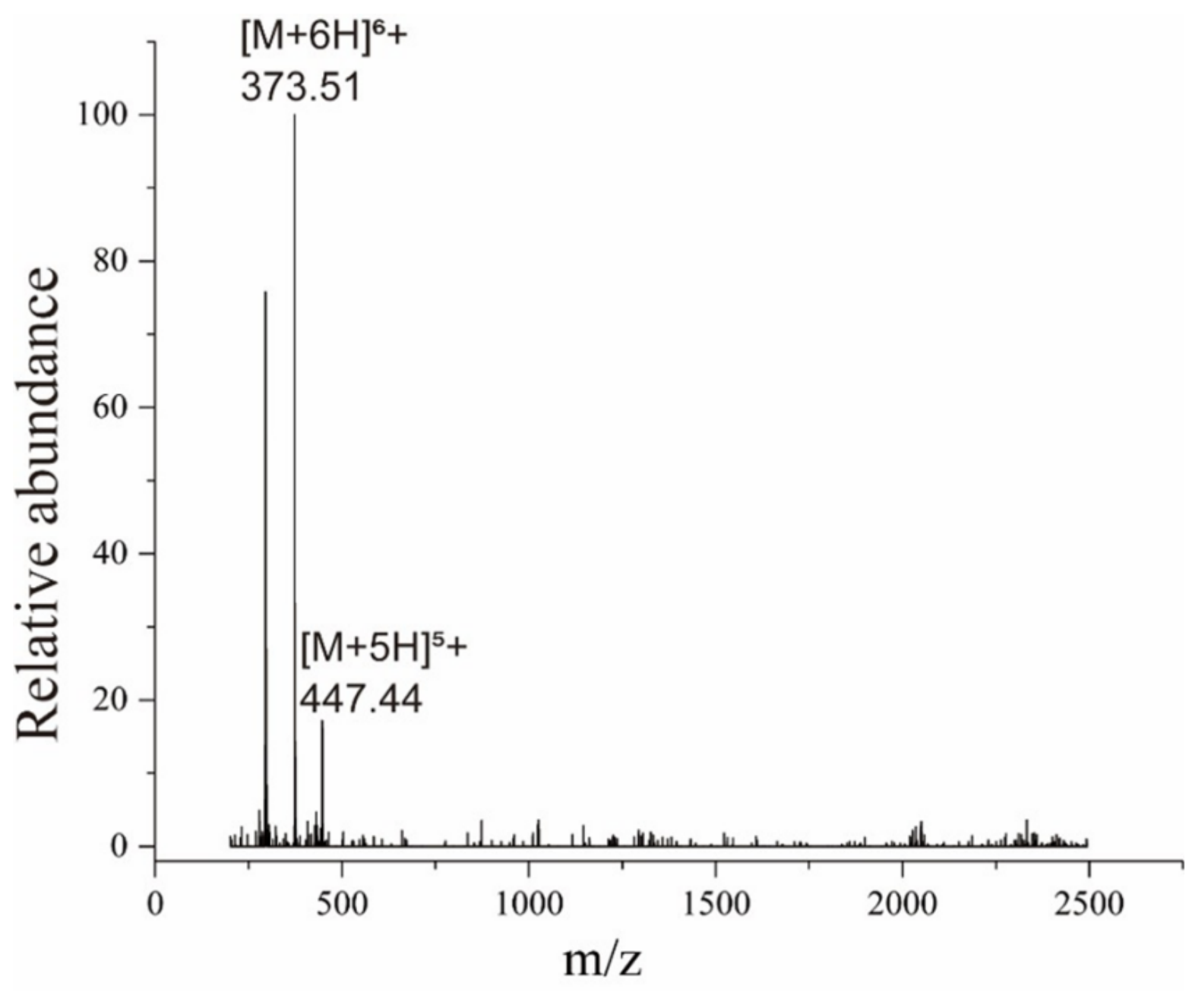

FIGURE S7 | (a) HPLC chromatogram of **BFG**. (b) ESI mass spectrum of the fraction collected at 23 min.

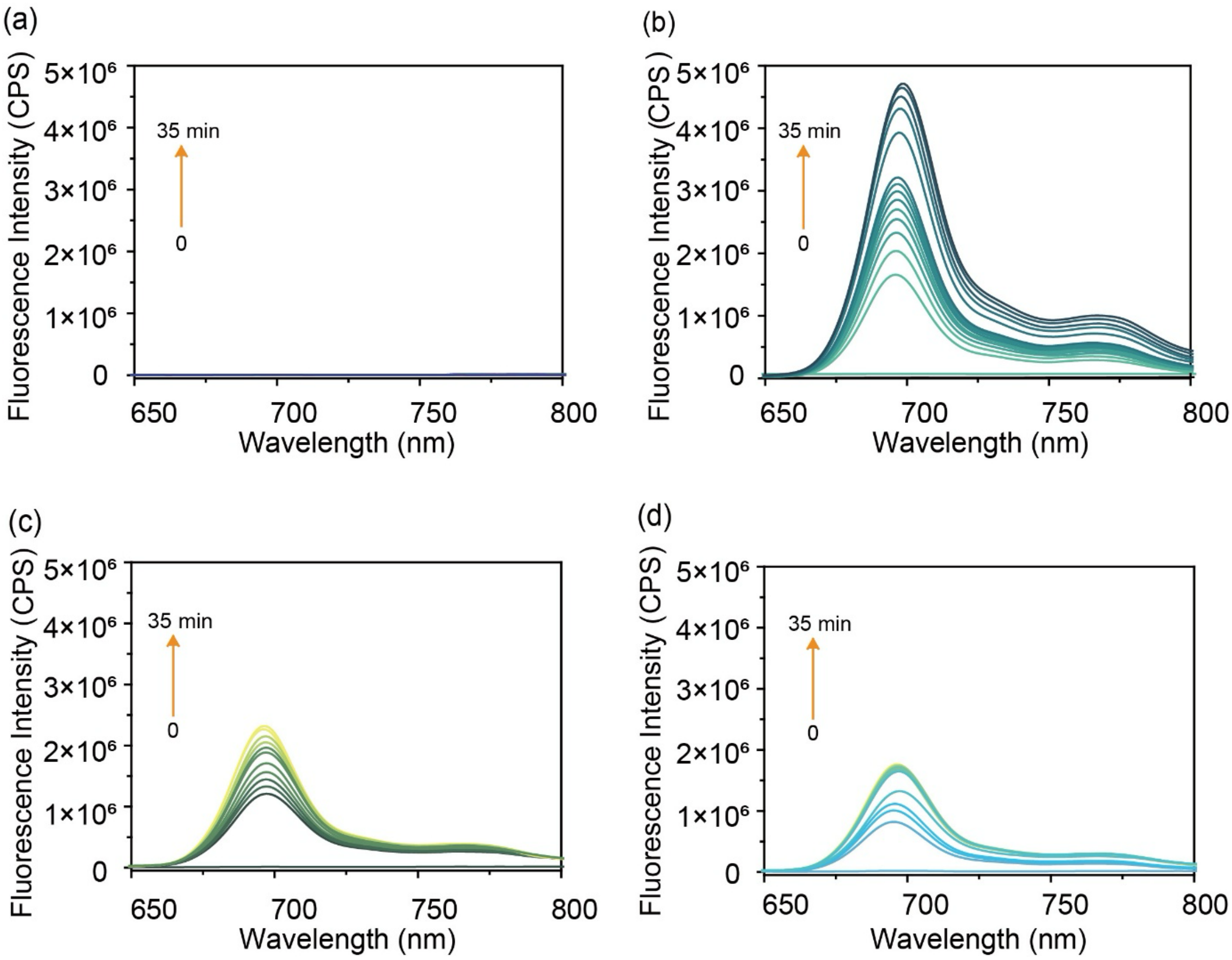

FIGURE S8 | Change in fluorescence spectrum of **PcNP** (at 5 μM of **Pc–Tz**) in deionized water in the (a) absence and presence of (b) **BF**, (c) **BG**, and (d) **BFG** (all at 120 μM) over a period of 35 min.

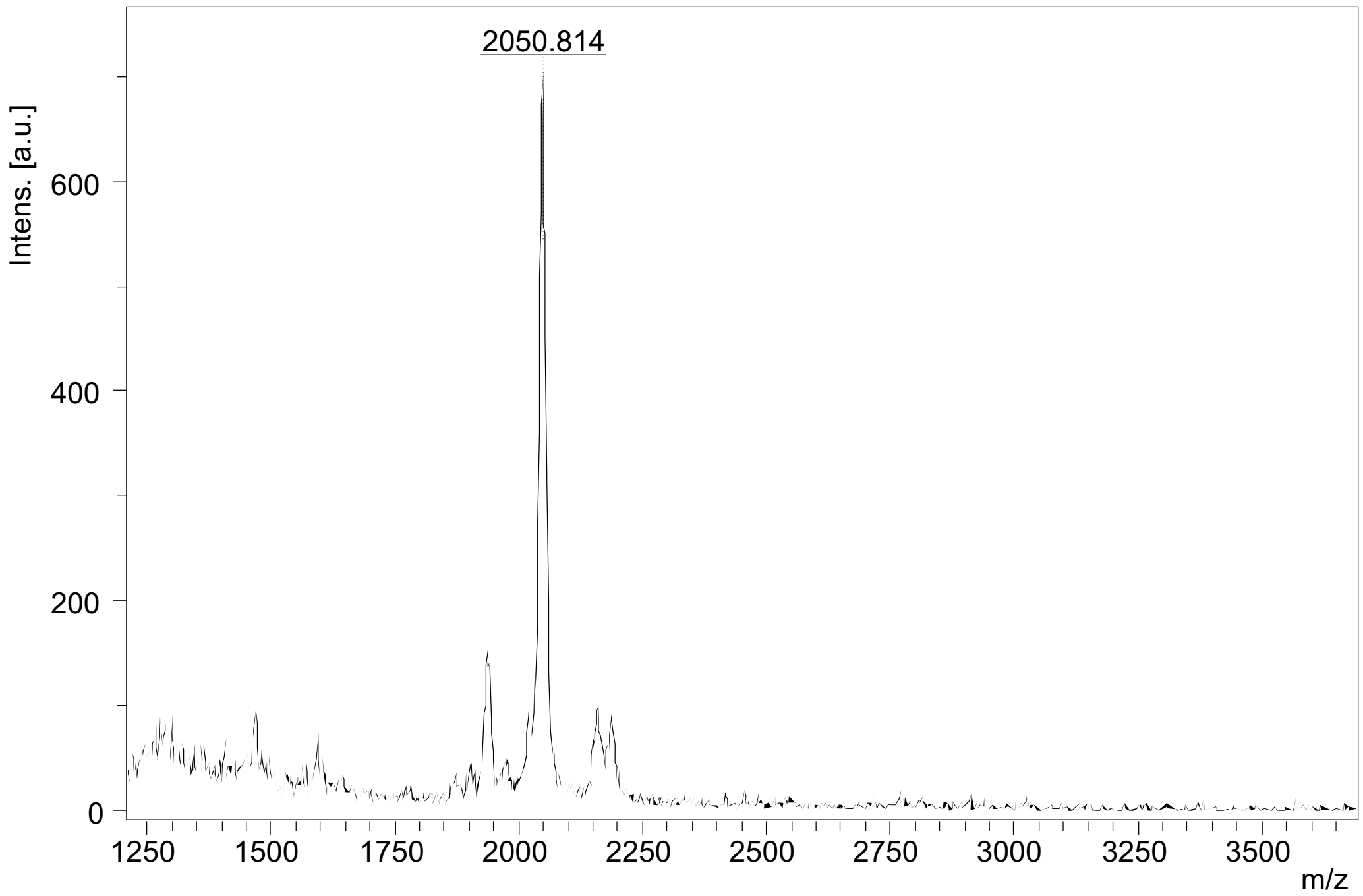

FIGURE S9 | MALDI-TOF mass spectrum of the reaction mixture after treating **PcNP** (at 5 μM of **Pc–Tz**) in deionized water with **BF** (120 μM) at room temperature for 35 min.

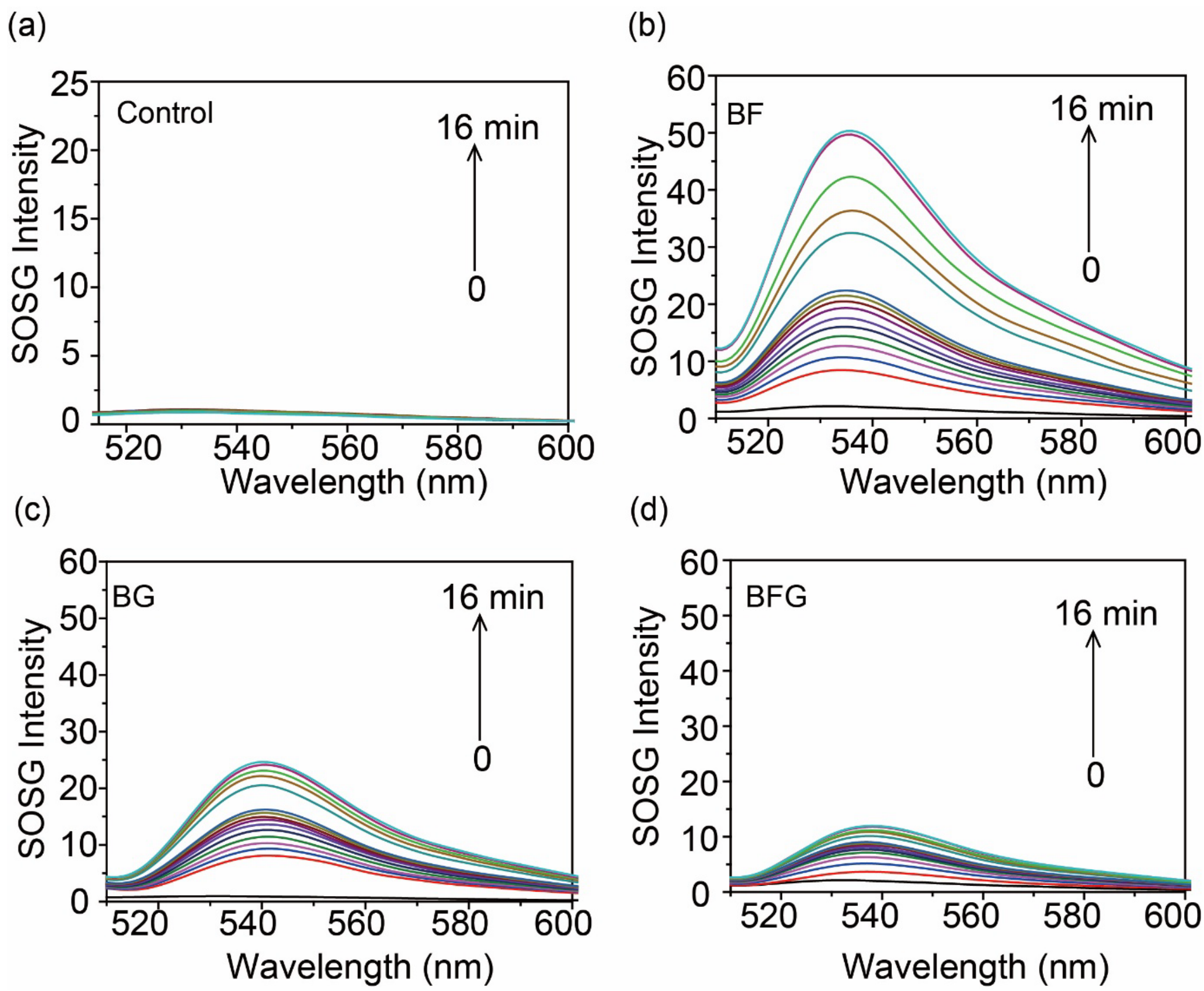

FIGURE S10 | Change in fluorescence spectrum of SOSG (10 μM) in deionized water containing **PcNP** (at 5 μM of **Pc–Tz**) (a) in the absence of a BCN derivative or after the treatment with (b) **BF**, (c) **BG**, or (d) **BFG** (all at 120 μM) for 35 min, followed by light irradiation (λ > 610 nm) over a period of 16 min. The fluorescence spectra were recorded at 1-min intervals.

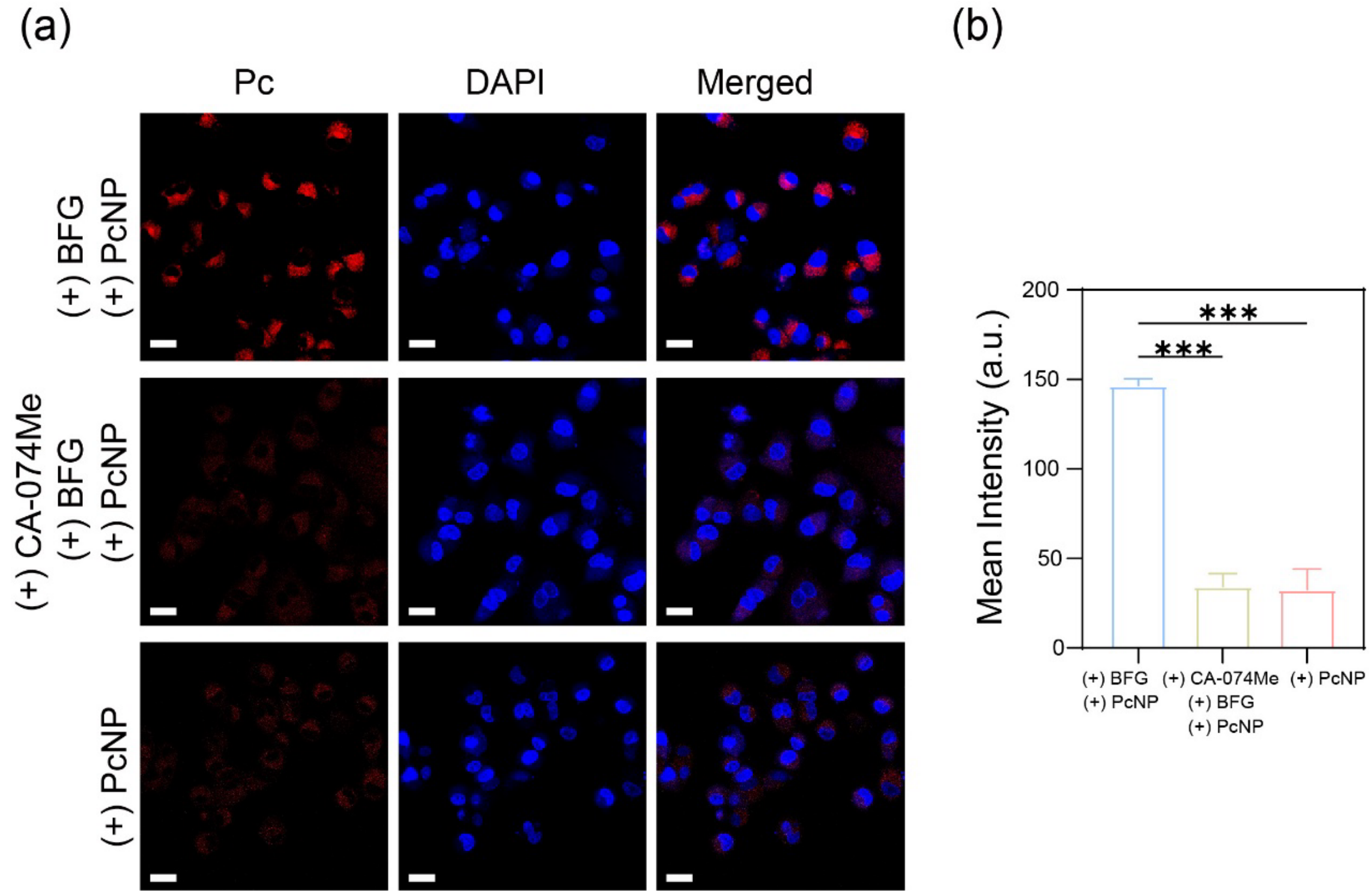

FIGURE S11 | (a) Confocal microscopic images of A549 cells after sequential incubation with **BFG** (120 µM, 4 h) and **PcNP** (at 5 µM of **Pc–Tz**, 1 h) with or without pre-treatment with CA-074Me (25 µM, 2 h). As a baseline control group, cells were incubated with **PcNP** (at 5 µM of **Pc–Tz**) only. The fluorescence of ZnPc is indicated in red, while cell nuclei were counterstained with DAPI (in blue). Scale bar: 20 µm. (b) Quantification of the mean intracellular fluorescence intensities for the three groups. Data are presented as mean ± SD of three independent experiments. Statistical significance was analyzed by an unpaired two-tailed Student's t-test (***$p < 0.001$).

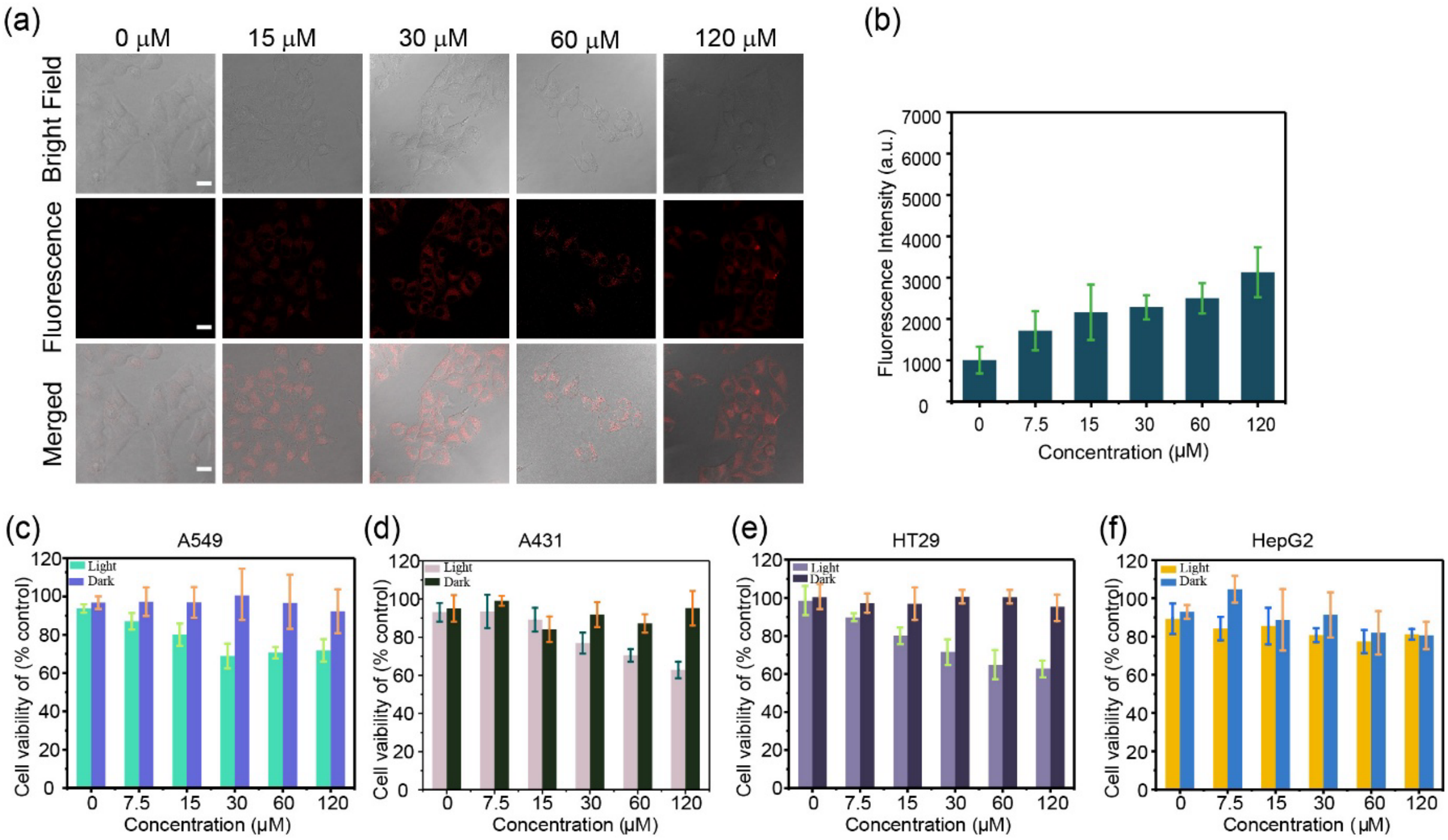

FIGURE S12 | (a) Confocal microscopic images of A549 cells after incubation with **PcNP** (at 5 μM of **Pc–Tz**) for 1 h with or without pre-incubation with different concentrations of **BG** (0–120 μM) for 4 h. Scale bar: 20 μm. (b) Intracellular fluorescence intensities under the above conditions determined by flow cytometry. Data are reported as mean ± SD of three independent experiments. Cytotoxicity of **PcNP** (at 5 μM of **Pc–Tz**) triggered by **BG** (120 μM) against (c) A549, (d) A431, (e) HT29, and (f) HepG2 cells, both in the dark and upon light irradiation (λ > 610 nm, 23 mW cm^{-2}) for 10 min. Data are reported as mean ± SEM of three independent experiments, each performed in quadruplicate.

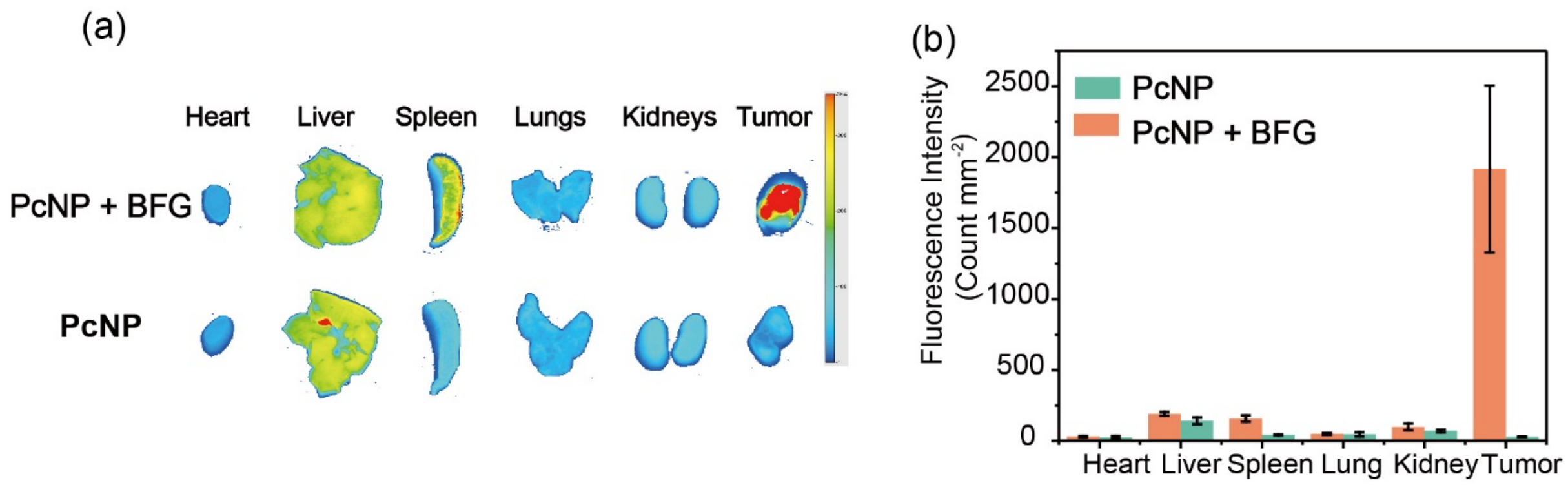

FIGURE S13 | (a) Representative ex vivo images and (b) quantified fluorescence intensities of the tumors and some major organs harvested at 78 h post-injection of **PcNP** for the two groups of mice (n = 3).

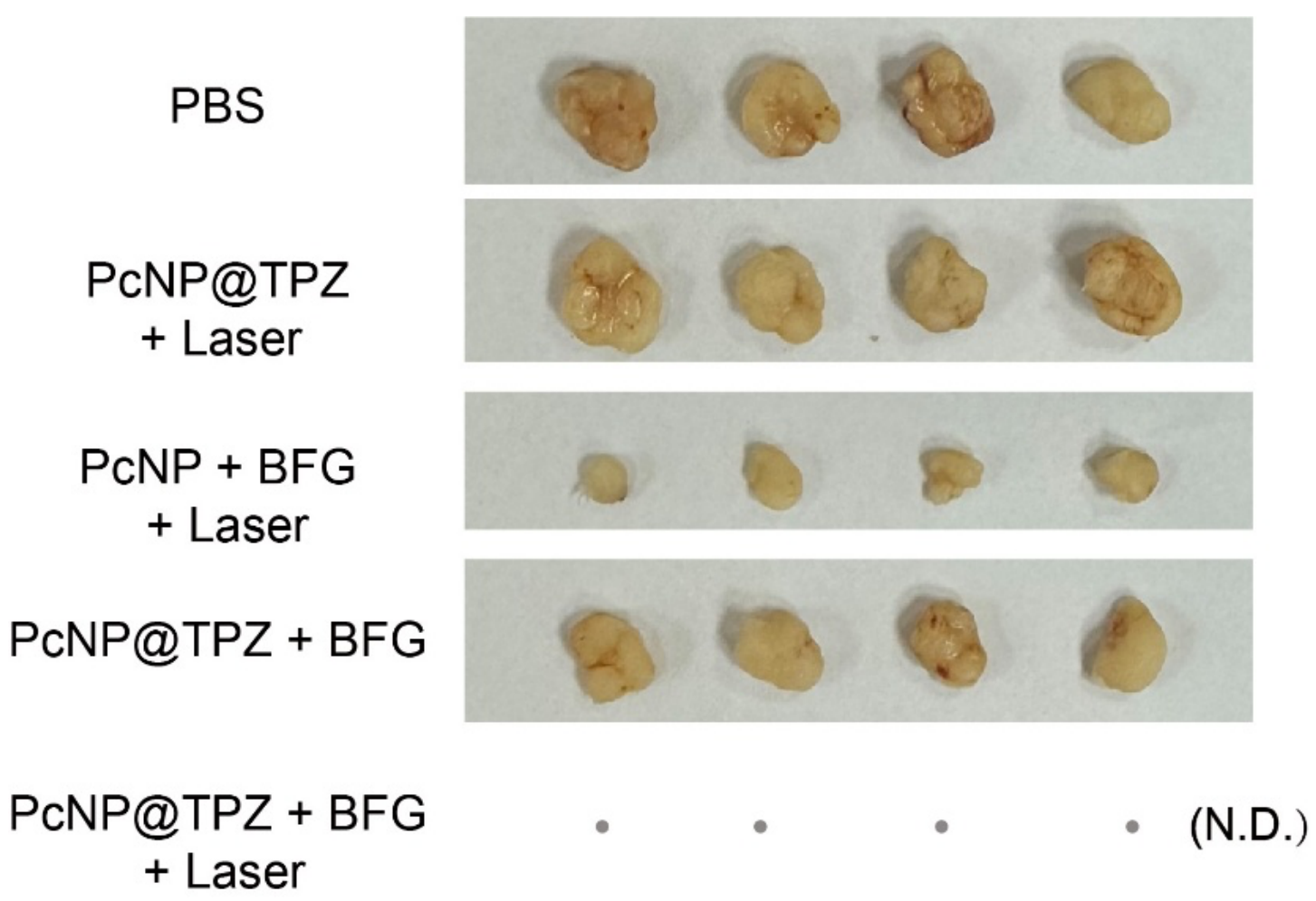

FIGURE S14 | Representative photographs of the tumors in the mice on Day 14 after different treatments.

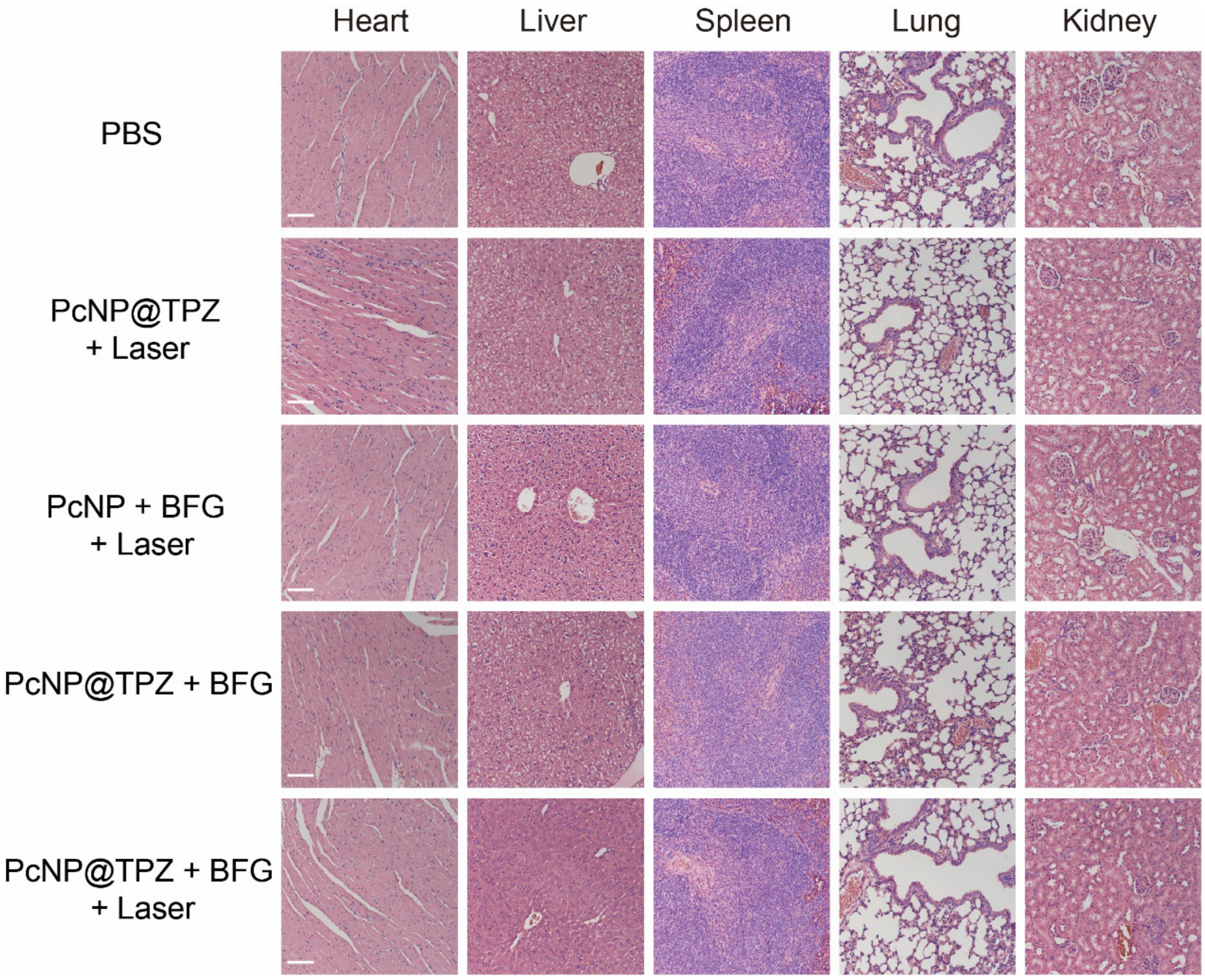

FIGURE S15 | Representative H&E-stained images of the major organs after different treatments. Scale bar: 200 μm.

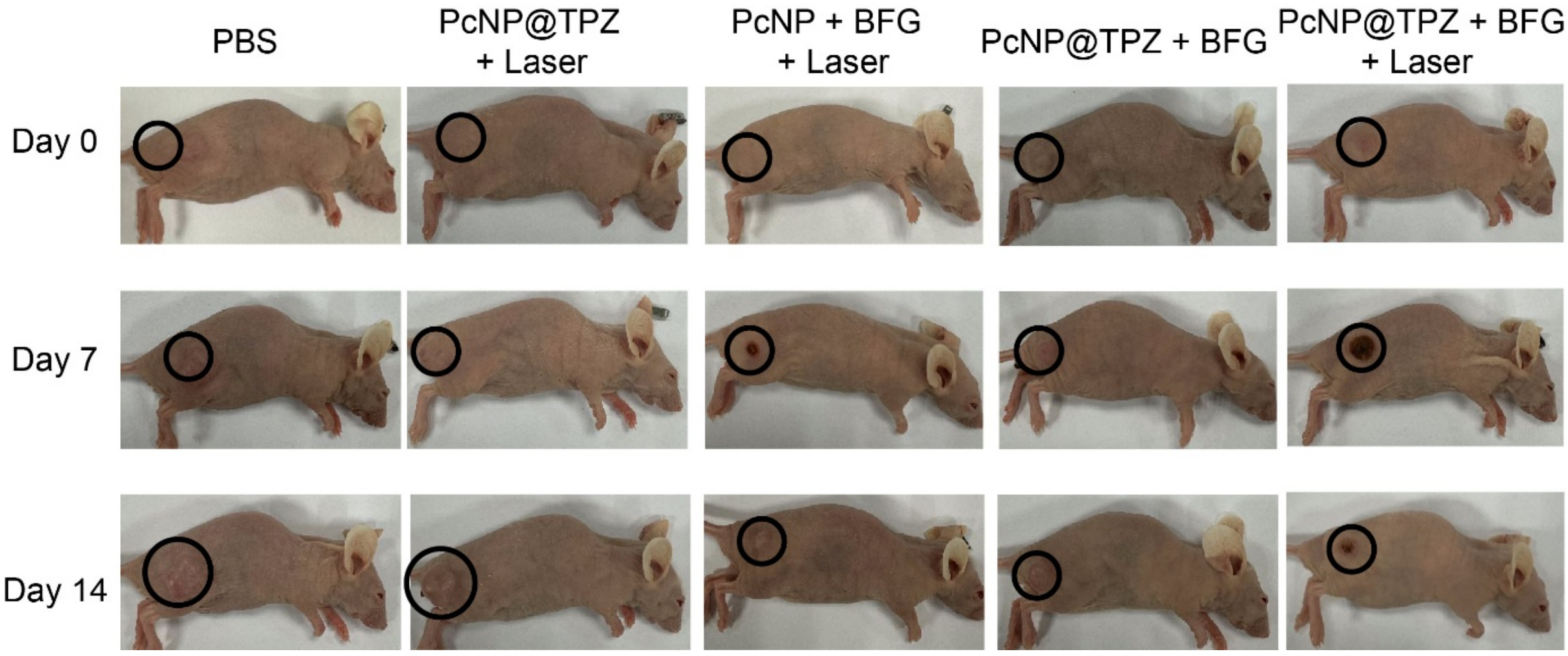

FIGURE S16 | Representative photographs of the mice during the course of different treatments.

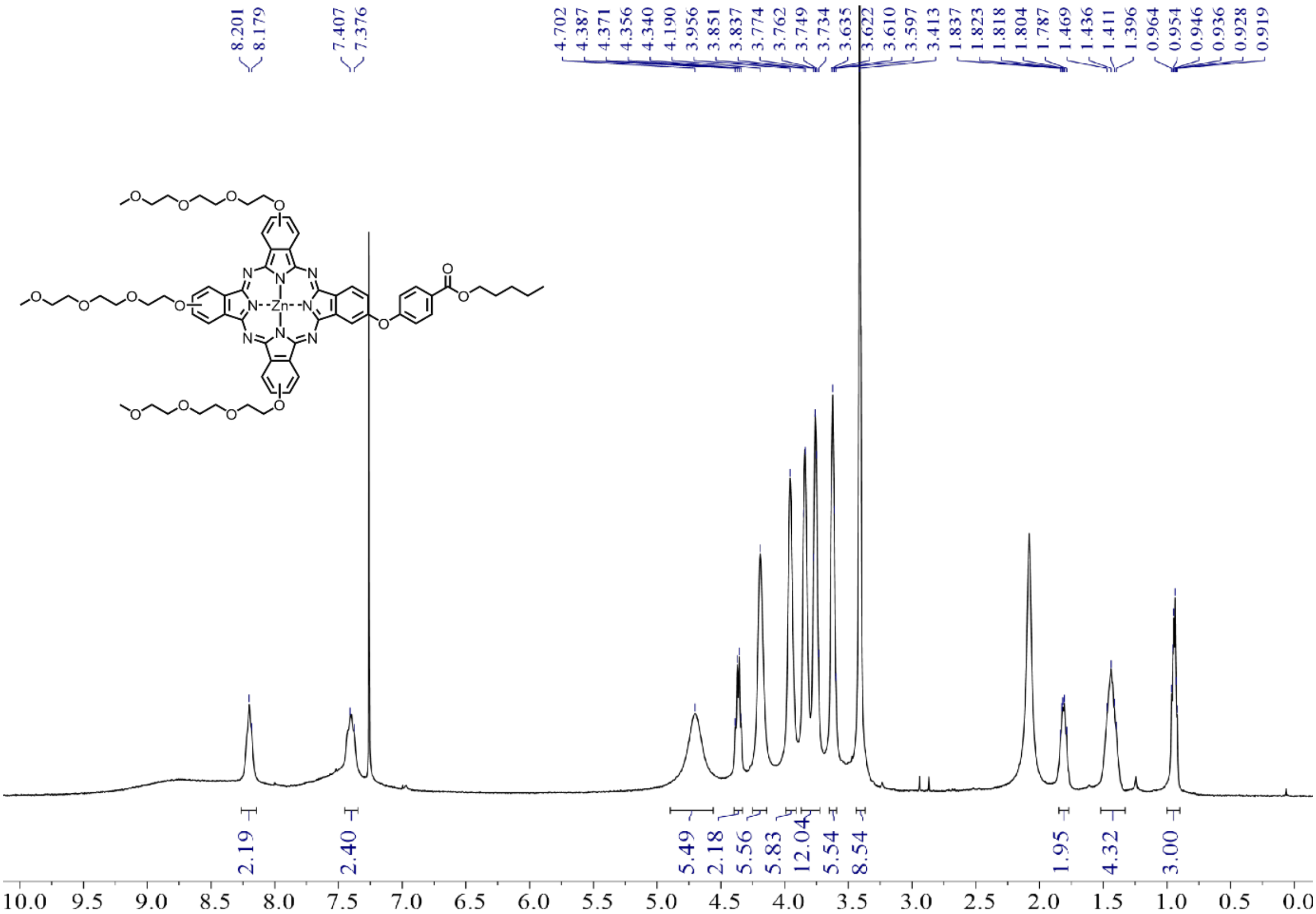

FIGURE S17 | ^{1}H NMR spectrum of **3** in $CDCl_3$ with a trace amount of pyridine-d_5.

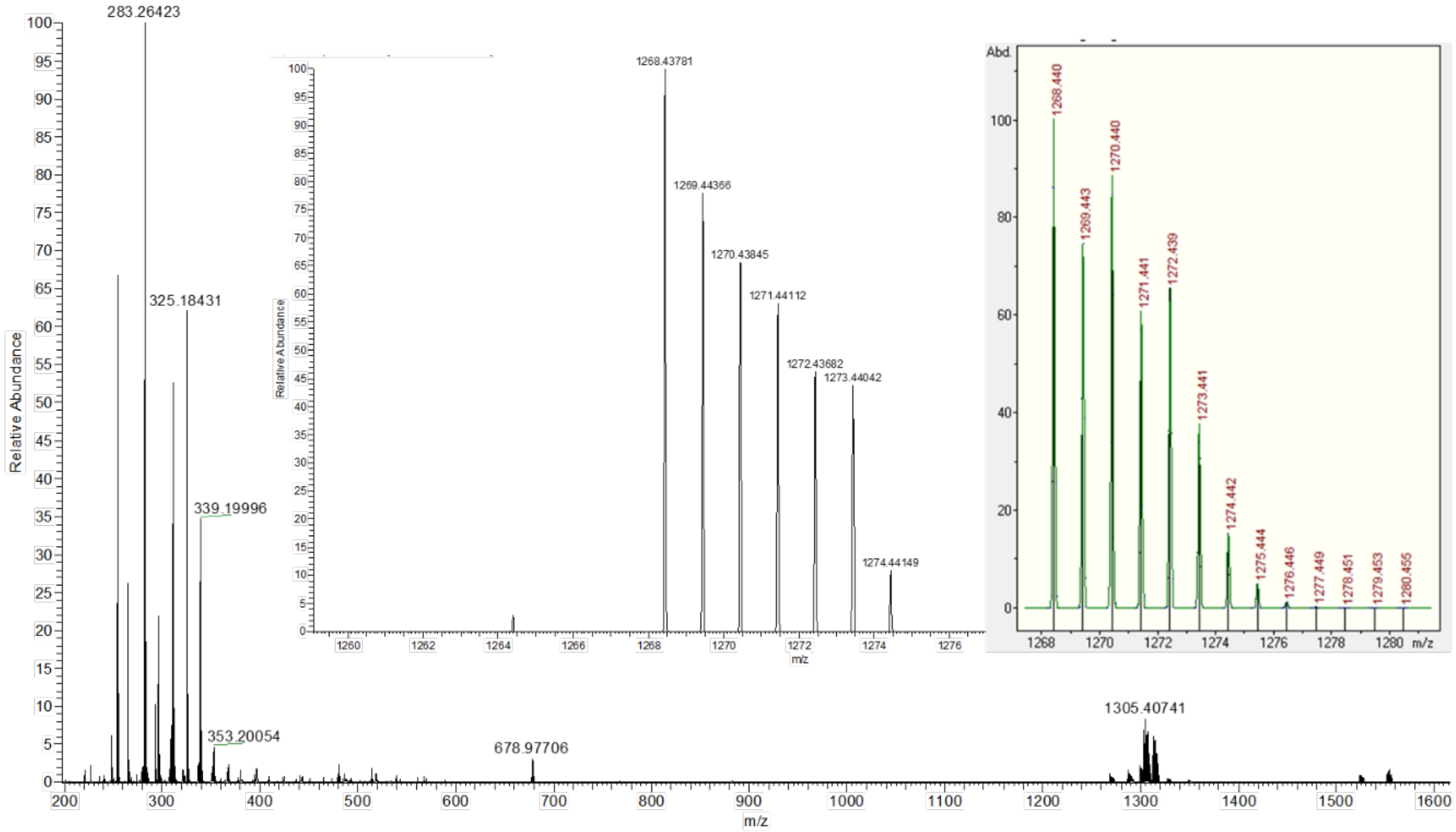

FIGURE S18 | High-resolution ESI mass spectrum of **3**. The experimental (left) and simulated (right) isotopic patterns of the molecular ion signal are shown in the inset.

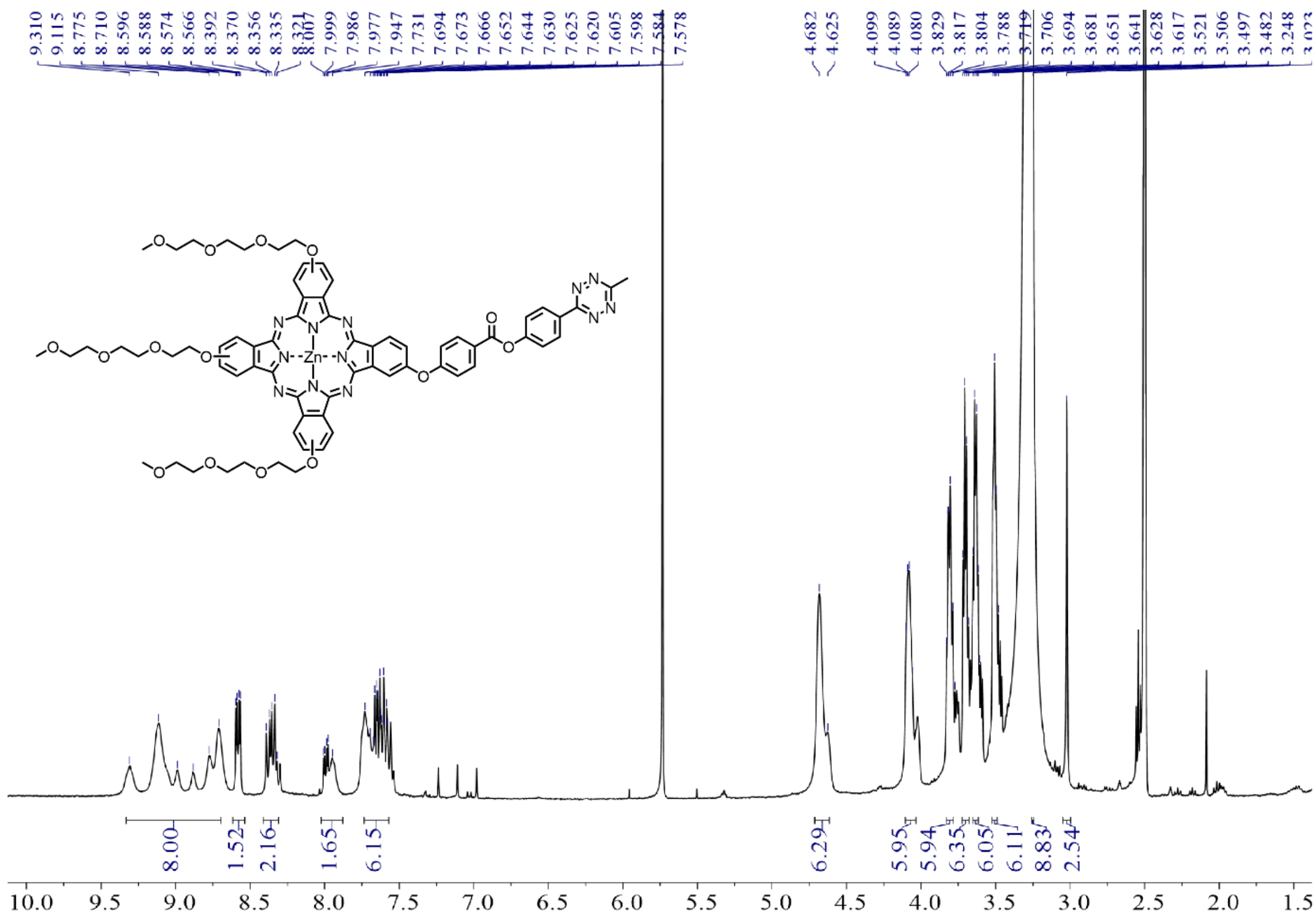

FIGURE S19 | ^{1}H NMR spectrum of **Pc–Tz** in DMSO-d$_6$.

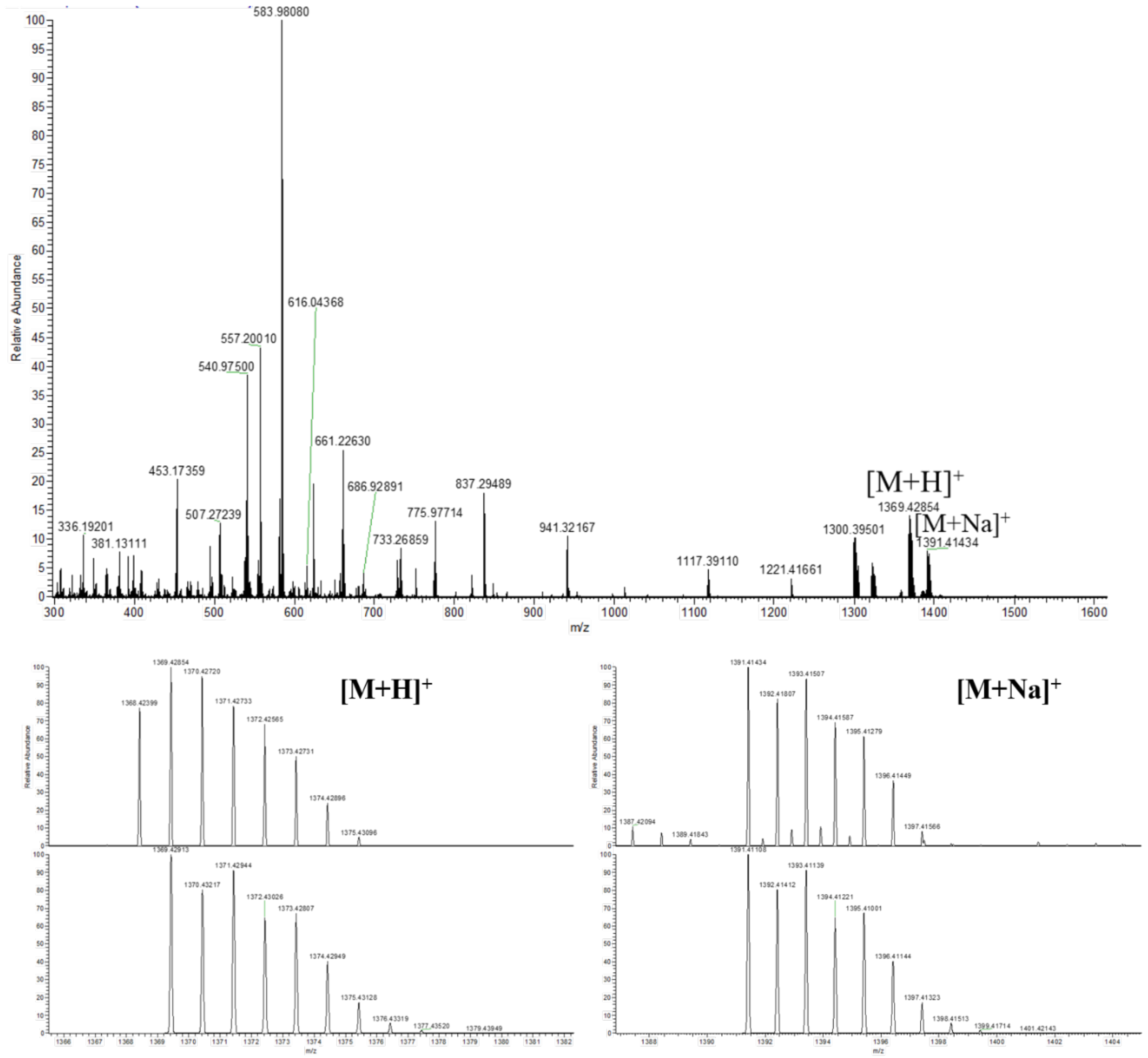

FIGURE S20 | High-resolution ESI mass spectrum of **Pc–Tz**. The experimental (top) and simulated (bottom) isotopic patterns of the protonated and sodiated molecular ion signals are shown in the lower left and right, respectively.
